# Survey of transcriptome analyses of hippocampal neurogenesis with focus on adult dentate gyrus stem cells

**DOI:** 10.3389/fcell.2025.1605116

**Published:** 2025-05-30

**Authors:** Laura Micheli, Maurizia Caruso, Giorgio D’Andrea, Daniel Volpe, Manuela Ceccarelli, Felice Tirone

**Affiliations:** 1 Institute of Biochemistry and Cell Biology, National Research Council, Rome, Italy; 2 Onco-Hematology, Cell Therapy, Gene Therapies and Hemopoietic Transplant, Bambino Gesù Children’s Hospital IRCCS, Rome, Italy

**Keywords:** transcriptomic analyses, RNA sequencing, adult neurogenesis, aging, dentate gyrus stem cells, self-renewal, gene expression profiling

## Abstract

Adult mammalian brains generate new neurons throughout life in two main niches, the dentate gyrus of the hippocampus and the subventricular zone, starting from neural stem cells (NSCs). Adult hippocampal neurogenesis is crucial for learning and memory and decreases during aging. As defined in mouse models, NSCs, which are prevalently quiescent, develop into proliferating progenitor cells, neuroblasts, and immature and mature neurons. Two visions for NSC self-renewal in the dentate gyrus have been proposed, one postulating persistent self-renewal, with cycles of rest and reactivation even in old age, and the other proposing a short-lived NSC model. Single-cell RNA sequencing and clonal studies, discussed in this review, have shed light on the developmental steps of neurogenic cells and the modality of self-renewal, revealing the presence in the adult dentate gyrus of NSC heterogeneous populations, one long-lived and another rapidly depleted at an early age. Another relevant question is whether adult neurogenesis occurs in humans. A few single-cell RNA-seq studies show that new neurons, with prolonged neuronal maturation, are continuously generated at low frequency from stem/progenitor cells, which results in the accumulation of immature granule cell neurons. This suggests an important role of these cells in human neurogenesis and hence interspecies differences in the neurogenic process dynamics. This review is focused on transcriptomic studies that have faced these and other NSC issues by analyzing developmental trajectories of neural cells and NSCs gene expression profiles in specific experimental settings of hippocampal neurogenesis, and also in mouse models with deletion or overexpression of specific genes to reproduce neural pathologies.

## Introduction

New neurons are continuously produced from stem cells during adulthood and throughout life in two distinct neurogenic niches—the dentate gyrus of the hippocampal region and the subventricular zone (SVZ) next to the ventricles (Kempermann et al., 2015; Lim and Alvarez-Buylla, 2016).

Adult neurogenesis in the hippocampus is critical for learning and memory, specifically for pattern separation - i.e., the capacity to distinguish between similar memory patterns - which is a function of dentate gyrus circuitry enhanced by the addition of new neurons to existing circuits (Aimone et al., 2011; Sahay et al., 2011; Farioli-Vecchioli et al., 2008).

In the dentate gyrus, stem cells with radial glia-like (RGL) morphology (Seri et al., 2001), called type-1 and localized in the subgranular zone (SGZ), express Glial Fibrillary Acidic Protein (GFAP), Nestin and Sex Determining Region Y-Box 2 (Sox2). Neural stem cells (NSCs) progressively develop into proliferating neural progenitor cells (NPCs), designated as type-2a (Nestin^+^/Sox2^+^), type-2b cells (expressing Nestin and doublecortin: Nestin^+^/DCX^+^) and neuroblasts (type-3, DCX^+^) (Filippov et al., 2003; Fukuda et al., 2003; Kronenberg et al., 2003; Steiner et al., 2006). Neuroblasts progress toward immature postmitotic granule neurons co-expressing DCX and NeuN (stage 5), and eventually become terminally differentiated neurons (stage 6) expressing calbindin and NeuN (Brandt et al., 2003; Steiner et al., 2004).

These maturation steps of adult dentate gyrus stem cells have been defined essentially by studying the mouse brain. Some critical issues have emerged that are actively investigated, namely, whether adult neurogenesis is present also in human hippocampus, with evidence in favor (Spalding et al., 2013; Boldrini et al., 2018; Seki et al., 2019; Tobin et al., 2019; Moreno-Jiménez et al., 2019; 2021) and against (Dennis et al., 2016; Sorrells et al., 2018; 2021), and whether the developmental trajectories of neurogenesis are the same in primates and mouse. Some studies attempt to answer these questions by analyzing the transcriptome of human and primate hippocampal neurogenesis in adults and during development, comparing it with the transcriptome of mouse neurogenesis. Moreover, there is debate over the model of neurogenesis - either continuous or displaying discrete developmental steps leading to different cell types - and also on the model of self-renewal of stem cells in the dentate gyrus, i.e., about whether the stem cell pool is depleted during aging or not. This latter question is related to the previous and concerns also the possibility to reactivate the quiescent stem cell by a neurogenic stimulus.

This review is focused on studies that have approached these questions by analyzing gene profiles in specific experimental settings of hippocampal neurogenesis, in hippocampus *in vivo* or in cell models from primates and mice, both in wild-type and in mouse models with deletion or overexpression of specific genes, in order to reproduce neural pathologies. The major focus of this review is on adult dentate gyrus stem cells. The studies analyzed have been identified through MEDLINE using the key words: “transcriptome”, “gene expression profiling”, “dentate gyrus stem cells”, “RNA sequencing”, “hippocampus neural stem cells” and “neurogenesis and gene”.

### Adult hippocampal neurogenesis in primates *versus* mouse–single-cell/single-nucleus sequencing

Recent studies sought to clarify the process of adult neurogenesis in humans and primates at the cellular and transcriptional levels. In general, advances in RNA-seq technologies have enabled analysis at single cell resolution of the transcriptome of cell populations involved in the adult hippocampal neurogenesis. Although neurogenesis is a process shared across species, gene expression patterns in primates do not completely overlap those of rodents. Moreover, the primate hippocampus has a longer maturation period of newly generated granule cells (GCs) and a higher percentage of immature dentate GCs, compared with rodents (see for review Li et al., 2023). We discuss here transcriptomic studies on neurogenesis in primates.

- Hao et al. (2022) analyzed about 200.000 cells and the corresponding transcriptomes from the adult macaque hippocampus and identified more than 30 cell populations comprising radial glia-like cells, i.e., neural stem cells (NSCs), which are also able to generate neurospheres *ex-vivo*, intermediate progenitor cells and neuroblasts, and compared them with mouse adult neurogenesis. Comparison with mouse single-cell transcriptomic data revealed that hippocampal cell types are conserved, although each neurogenic cell population exhibits divergences in gene expression profile between rodents and primates. A peculiar difference, as judged by cells labeled by DCX and Prospero homeobox 1 (*Prox1*; a gene expressed in type-2b progenitor cells, neuroblasts and neurons; Steiner et al., 2008) was that the adult macaque hippocampus has a significantly higher proportion of post-mitotic immature granule cells (imGCs; i.e., immature neurons) than the adult mouse hippocampus.
- In another study by single-nucleus RNA-seq of the hippocampus from young and aged monkeys (macaque), Zhang et al. (2021) identified 12 cell types based on their unique gene-expression signatures, including the neurogenic lineage cells comprised of NSCs, transiently amplifying progenitor cells (TAPCs; i.e., type-2 and type-3 cells), immature neurons, and excitatory and inhibitory neurons. It was found that the most expressed (upregulated) genes in stem cells of aged hippocampus included *LMNTD1*, *PATCHED1* and *PATCHED2*, *GSAP*, *SORCS3*, while the most upregulated in TAPCs resulted *MEIS2*, *CDH13*, *FUGN, GRIP2*, *CLMP*, thus with some difference compared to the mouse markers of age (Ibrayeva et al., 2021, see below); the genes most upregulated in imGCs included *CAMK2D*, *MTUS2*, *ARMH1*, *ZNF804B.* Altogether, the findings identified key aging-related differentially expressed genes in multiple cell types that may underly the phenotype of hippocampal aging, characterized by increased heterochromatin erosion, genomic instability, cytosolic Amyloid-beta aggregation, inflammation and senescence and decreased neurogenesis. Among the 12 identified cell types, the most affected by aging were TAPCs and microglia, with impaired TAPC neurogenesis and high pro-inflammatory responses in the aged microglia.
- Interestingly, in another study by single-nucleus RNA-seq of macaque hippocampi across the lifespan, Wang et al. (2022) found a *continuum* of cell populations from adult NSCs to immature and mature granule cells, and in particular they identified 29 genes expressed in NSCs but undetectable in the dentate gyrus of mouse hippocampus. For instance, ethanol phosphate phospholipase (*ETNPPL*) was expressed in actively proliferating macaque SGZ stem cells, co-expressed with *SOX2*, but not in mouse, thus representing a primate-specific NSC marker. The authors analyzed also aged human hippocampi and found proliferating NSCs and immature granule neurons.

In human the existence of newly generated immature dentate gyrus granule cells is under debate (Gage, 2019; Sorrells et al., 2021; Moreno-Jiménez et al., 2019; Kempermann et al., 2018), being relevant for the issue of the presence of adult neurogenesis, since increasing evidence in mouse is attributing a role in the adult hippocampal plasticity to imGCs, which are functionally different from mature neurons (Schmidt-Hieber et al., 2004; Ge et al., 2007; Marín-Burgin et al., 2012). In fact, three reports could not identify imGC specific populations in human single-nucleus RNA-seq datasets using the conventional unsupervised clustering method (Ayhan et al., 2021; Habib et al., 2017; Franjic et al., 2022).

- Recently, Zhou et al. (2022) performed single-nucleus RNA-seq of human hippocampi from infant, juvenile, adult and aging brain specimens in an effort to identify and quantify imGCs (identified as DCX^+^ and PROX1^+^ neurons) and to define their gene signature at different ages. Indeed, they were able to identify imGCs in every hippocampal specimen across all ages through a supervised machine learning-based approach, without using established markers, i.e., given a training set of cell prototypes, AI extracted *de novo* features to quantify the similarity of each individual cell to each prototypical cell type. They also found by immunohistochemistry that stem/neural progenitors are generated in adult human dentate gyrus. Age-dependent changes in gene expression were detected by aligning human imGC transcriptomes on a pseudo-age trajectory using the software Monocle, indicating dynamic molecular properties of human imGCs across the lifespan. Furthermore, Zhou et al. found in imGCs enrichment of genes related to nervous system development (e.g., *NEUROD1*, *BHLHE22*), ion transport (e.g., *FXYD7*, *KCNQ5*), and neuron projection development (e.g., *SEMA6D*, *NR2F1*). Only 15% of these genes overlapped with mouse orthologs, with evident interspecies differences. Very low numbers of GC fate-specific proliferating neural progenitors were detected in the adult human hippocampus by immunohistology, indicating low frequency of the *de novo* generation of imGCs. However, a birth dating study performed on cultured surgical specimens demonstrated the capacity of the adult human hippocampus to generate new neurons. Based on the above data, the authors suggested that new neurons are continuously generated at low frequency from stem/progenitor cells, but exhibit protracted neuronal maturation resulting in the accumulation of imGCs at any given time in the adult human hippocampus (Zhou et al., 2022).
- Another recent study investigated adult hippocampal neurogenesis in *post-mortem* dentate gyrus from infant, adolescent, and middle-aged males by combining spatial transcriptomics and multiplexed fluorescent *in situ* hybridization (Simard et al., 2024). Similarly to what was observed by Zhou et al. (2022), very few cells expressing NSC and proliferative markers were detected in the dentate gyrus from childhood to middle age, whereas the number of imGCs was substantially elevated. Few NESTIN^+^SOX2^+^ cells were found in SGZ, and also the low expression of PCNA observed in all samples suggests that the majority of NSCs in dentate gyrus proliferate mostly prenatally. Although the issue whether imGCs are newly generated or not remains unanswered, their presence in adult dentate gyrus, demonstrated by the transcriptomic studies mentioned above and other previous studies (Boldrini et al., 2018; Tobin et al., 2019; Moreno-Jiménez et al., 2019), suggests an important role of this cell population in driving adult hippocampal plasticity throughout human life in physiological and pathological conditions.
- In another study, single-cell RNA sequencing was used to identify hippocampal cell types and their molecular features during human brain development (Zhong et al., 2020). At gestational weeks 16–27, Zhong et al. identified 47 cell subtypes and their developmental trajectories and established a parallel between the molecular features of the human hippocampus gestational weeks 16–20 and those of the mouse at postnatal days (P) 0–5, indicating a conserved developmental mechanism. However, in agreement with Zhou et al. (2022), Zhong et al. (2020) found that the molecular characteristics of the mouse hippocampal region at P0-P5 and the human hippocampal region during gestational weeks 16–20 present variations in gene expression between the two species.

See Table 1 for section summary.

**TABLE 1:** Primate dentate gyrus transcriptomic studies.

Aim	Conclusions/key findings	Genes regulated/cell types	RNA-seq details	Marker usage/Methods	References
Investigation on the extent of neurogenesis in adult primates	Identified 34 cell populations, including RGLs, IPs and neuroblasts. Comparison with mouse adult neurogenesis	Higher ratio of immature GCs in adult macaque hippocampus (DCX+ and Prox1+ cells) compared to mouse. Identified RGL cells (GFAP^+^HMGB2^-^)	scRNA-seq of 200.000 cells from the adult macaque hippocampus	Standard markers of DG neurogenesis. *Ex-vivo* analysis of neurosphere clones	Hao et al. (2022)
Study of aging in hippocampal neurogenesis in monkey (macaque)	Identified 12 cell types; aging most affects TAPC and microglia, impairing TAPC neurogenesis and increasing pro-inflammatory responses in aged microglia	Aging-related differentially expressed genes: *LMNTD1*, *PATCHED1 AND PATCHED2, GSAP*, *SORCS3* in NSCs (associated to phenotypic and transcriptomic signatures of hippocampal aging)	snRNA-seq: isolated nuclei from hippocampi from eight young (4**–**6 years old) and eight aged (18**–**21 years old) macaque	Standard markers to identify cell types	Zhang et al (2021)
Study of hippocampal neurogenesis in macaque vs mouse and aged human	Identified 29 genes expressed in macaque NSCs but undetectable in the DG of mouse hippocampus	ETNPPL is primate-specific NSC marker: expressed in proliferating Sox2-positive macaque SGZ NSCs, but not in mouse	snRNA-seq of 132.000 from adult macaque and 22.000 cells from aged human hippocampus	Monocle 3 used to construct the macaque and human adult neurogenic trajectory	Wang et al. (2022)
Identification of the gene signature of human immature DG granule cells at different ages	ImGCs (DCX^+^Prox1^+^ neurons) mature slowly and are more abundant in human than in mice. NPCs are generated also in adult human DG	Enrichment in imGCs of genes related to nervous system development (*NEUROD1*, *BHLHE22*), ion transport (*FXYD7*, *KCNQ5*), and neuron projection development (*SEMA6D*, *NR2F1*). Only 15% of these genes overlaps with mouse orthologous	scRNA-seq of human hippocampus from infant and adult brain specimens	Supervised machine learning approach to identify ImGCs Age-dependent changes monitored using Monocle software by aligning human imGCs transcriptomes on a pseudo-age trajectory	Zhou et al. (2022)
Study of DG in infant, adolescent, and middle-aged males	Few cells expressing NSC and proliferative markers detected in the DG from childhood to middle age; high number of imGCs	Low expression of NSC marker (NESTIN and SOX2) and of PCNA suggests that the majority of NSCs in DG proliferate prenatally	Spatial transcriptomic of frozen DG from human (male)	Deconvolution analysis to determine the gene expression profile of specific cell types. Combined spatial transcriptomics and multiplexed fluorescent *in situ* hybridization	Simard et al. (2024)
Identification of cell subtypes and developmental trajectories of human hippocampus	Gene expression of hippocampus in mouse during postnatal days 0–5 and in human during gestational weeks 16–20 show inter-species variation	Identified 47 cell subtypes and their developmental trajectories at months 4–7. Established a parallel between human gestational weeks 16–20 and P0-P5, indicating a conserved developmental mechanism	scRNA-seq from human hippocampus at gestational weeks 16–27	Standard markers and gene ontology (GO) of differentially expressed genes to identify cell identity	Zhong et al. (2020)

Abbreviations: DG, dentate gyrus; GC, granule cells; ImGC, immature GC; IP, intermediate progenitor; NSC, neural stem cell; RGL, radial glial like; sc/snRNA-seq, single-cell/single-nucleus RNA, sequencing; TAPC, transiently amplifying progenitor cell.

### Mouse hippocampal neurogenesis - single-cell or bulk RNA sequencing from isolated dentate gyrus

Two theories about the mechanism of stem cell self-renewal in the SGZ of the adult dentate gyrus have been put up in the past years, in mouse models; the first postulates recurrent stem cell self-renewal, while the second suggests a “disposable stem cell” model. According to the first paradigm, a quiescent stem cell that has been physiologically awakened may undergo many asymmetrical divisions, giving rise to neurons or astrocytes, or may undergo symmetric division. After either way, stem cells may go back to being quiescent and can be activated again later (Bonaguidi et al., 2011; Ceccarelli et al., 2020). Consistently, several studies indicated that some NSCs return to quiescence in both the SGZ and the SVZ, which may account for the preservation of the stem cell pool and neurogenesis in old age (Urbán et al., 2016; Obernier et al., 2018; Pilz et al., 2018). In contrast, the “disposable stem cell” hypothesis states that once activated the stem cell divides asymmetrically several times, and finally terminally differentiates into an astrocyte or a neuron, with a consequent depletion of the stem cell pool (Encinas et al., 2011).

More recently, these two views have been brought together by the recognition of the role that aging plays in NSC self-renewal. Indeed, in the early postnatal stages, the “disposable” model would be prevalent, with rapid replication that in the end would lead to NSC pool depletion. Conversely, in later stages and during aging, NSCs would replicate in a way that privileges the pool’s conservation through a gradual pattern of self-renewal; this age-related change coincides with a transition from a population of NSCs that divides frequently to a more quiescent population (Harris et al., 2021; Ibrayeva et al., 2021; Martín-Suárez and Encinas, 2021). The research by Ibrayeva et al. (2021) is instructive since it demonstrates that there are heterogeneous populations of NSCs, as Nestin-positive NSCs have a longer lifespan than NSCs expressing the pro-activation gene *Ascl1* (Achaete-Scute bHLH factor 1) because they divide less often with an increase in quiescent cells as they age (see below). Several studies are in line with this view of the existence of NSCs subpopulations with different dynamics of activation and self-renewal over time.

Here we summarize transcriptomic studies that analyze neurogenesis in mouse dentate gyrus during development, early postnatal life, adulthood and aging. They point to elucidate the origin and the characteristics of adult NSCs and finally their behavior during aging.

- The study of Hochgerner et al. (2018) is focused on the definition of the relationship between developmental and adult neurogenesis in dentate gyrus. They show, by using single-cell RNA-seq analysis, that late embryonic, early postnatal and adult neurogenesis in the mouse dentate gyrus display conserved gene regulation. Authors propose a model of neurogenesis with well-defined cell types and transitions in perinatal and adult mice - rather than a continuous process - with long-lived self-renewing RGL cells (i.e., NSCs), which are predominantly quiescent, even during development. In particular, they observed that at postnatal week 2, RGL cells shift from an embryonic to an adult transcriptomic profile. Hochgerner et al. (2018) found that top enriched markers in RGL cells include *Id3*, *Sox9*, *Hes1*, *Tshz2*, *Hopx*, *Hes5*, *Tfap2c*, *Cox4i2*, *Rgs5*, *Rhcg*, and *Ascl1*; the absence of expression of cell cycle genes such as *Cdk1* and *Top2a* indicates that these NSCs are predominantly quiescent. The RGL cells were the most long-lived cells, and in their early neurogenesis stage were in a quiescent state specifically marked by expression of a very small number of genes, including *Rhcg*, *Vnn1*, and *Lpar1.* When NSCs become activated, the expression of some of the above genes increases in actively cycling progenitor cells (chiefly *Tfap2c*, *Ascl1*, *Sox9*, *Hopx*) in parallel to an increase of *Cdk1* and many other cell cycle genes. Notably, the vast majority of RGL cells that entered the cell cycle became progenitor cells expressing neurogenic factors such as *Neurod1*, *Neurod2*, *Neurod4*, and *Eomes*, during their sequential progression to neuroblast stages, whereas a small proportion of cells retained gene expression features of RGL cells with low or no expression of neurogenic factors. Altogether, this suggested that the population of RGLs could undergo a continuous and sustained self-renewal process by occasional proliferation without differentiation or by occasional asymmetric division.
- The study by Berg et al. (2019) used the *Hopx*-*CreER* ^*T2*^ mouse line to perform clonal lineage-tracing, population fate-mapping and transcriptome analyses of dentate gyrus Hopx^+^ neural progenitors. The authors found that the gene *Hopx* is expressed in quiescent RGL cells that retain the capacity to re-enter the cell cycle in the adult dentate gyrus, and that it also labels neural precursors and their progeny from an early embryonic stage to adulthood. Shin et al. (2015) also indicated that in the adult mouse Hopx labels quiescent NSCs. Collectively, the results identified a common Hopx^+^ neural precursor population that continuously contributes to embryonic, early postnatal and adult neurogenesis giving rise to granule neurons and adult RGLs in the dentate gyrus. Thus, Berg et al. (2019) propose the idea that dentate neurogenesis occurs as one continuous process throughout development from embryonic stage to adulthood, with some difference from the view of Hochgerner et al. (2018), which proposes the existence of a set of clearly defined, rather than continuous, cell types and transitions, with the most long-lived cells being the RGL cells from embryonic to adult dentate gyrus. For example, Berg et al. (2019) show that the expression of cell cycle (e.g., *Cdk1*, *Prc1*) and chromatin modification genes (*Smarcad1*, *Trim28*) was elevated at embryonic and postnatal stages, and then decreased in the adult stage. In contrast, cell surface signaling (e.g., *Nrxn1*, *Serpine2*) and lipid metabolism genes (e.g., *ApoE*) were gradually upregulated over development. Moreover, NSCs expressed some genes throughout the whole embryonic, early postnatal and adult period (*Vim*, *Sox9, Prom1*, *Pax6 and Hes1*). Functionally, there is evidence that Hopx favors apoptosis of NSCs by inhibiting the pro-survival serum responsive (*SRF*) transcription factor (De Toni et al., 2008).
- Another study showed by single-cell RNA-seq and by immunohistochemistry in the dentate gyrus of a *DCX-DsRed* transgenic mouse that Hopx labels weakly DCX-positive neuroblasts and is maximally expressed in NSCs (Gao et al., 2017).
- In a more recent report, the groups of Song and Bond (Jimenez-Cyrus et al., 2024) further investigated how the adult NSC pool is established by defining through single-cell RNA-seq the molecular cascade underlying NSC development in the early postnatal mouse dentate gyrus, taking advantage of the genetic marking of Hopx-expressing NSCs. They identified two sequential steps, first a transition to an immature quiescent state (between P3 and P7) followed by further maturation toward an adult state (between P7 and P14), with an autophagy burst before NSC quiescence attainment and increase of cellular reactive oxygen species along NSC maturation. Authors found distinct gene networks underlying each step of NSC development into an adult state. For instance, they observed a downregulation of genes such as *Nfix*, *Top2a*, *Rps3 and Sox11* during progression from the dividing state to an early quiescent state (named q1), and a parallel upregulation of genes such as *Npas3*, *Hopx and Sox9*.
- A previous study of Shin et al. (2015) defined the trajectories of quiescent and activated NSCs through the analysis of single-cell transcriptomes from quiescent NSCs and their early progeny, isolated from adult mouse hippocampus as *Nestin-CFP*-labeled cells. Authors identified cell types through analysis of known expression markers and used a software named Waterfall to reconstruct the continuous process of adult neurogenesis at single-cell resolution using adult neurogenesis as model. They used the expression profiles of few developmental genes to orient the possible trajectory of transcriptomic progression and then introduced pseudotime to define the relative location of each cell on the total path length. In this way Shin et al. identified the top 1000 positively- and 1000 negatively-correlated genes with pseudotime (UP^1000^ and DOWN^1000^), representing NSC-enriched genes up- and downregulated during activation and neurogenesis. Out of these genes, Shin et al. identified 41 transcription factors downregulated and 42 upregulated during hippocampal neurogenesis, including known regulators of neurogenesis, as *Sox2*, *Sox9*, *Fos* and *Id3* (all downregulated), *Sox4*, *Sox11* and *p53* (upregulated), and transcription factors less studied in adult neurogenesis such as *Dbx2*, *Id4* (downregulated), or *N-myc* (upregulated), or also paralogs to neurogenesis-related genes, such as SWI/SNF-related Brg1/Smarca4 associated factors. Interestingly, it turned out that the onset of NSC activation was associated with the downregulation of genes related to cell adhesion and various signaling pathways, namely, Notch signaling, GABA, BMP, MAPK and Calcium. Concurrently, genes involved in fatty acid oxidation and sphingolipid metabolism were enriched in quiescent NSCs but downregulated upon activation, which is consistent with literature reporting a metabolic shift from fatty acid oxidation to *de novo* lipid synthesis during activation of NSCs (Knobloch et al., 2013; 2017). Additionally, during NSC activation and at the onset of neurogenesis, some glycolysis genes decreased significantly, i.e., *aldolase A*, *aldolase C*, and *Ldhb*. All this suggested, according to Shin et al. (2015), that once activated, NSCs “shunt their capacity to respond to external regulation”.
- In an interesting study Borrett et al. (2022) compared the transcriptional trajectories of mouse NSCs in the SGZ of dentate gyrus and in the SVZ, using single-cell RNA sequencing data (by Hochgerner et al., 2018 and by Borrett et al., 2020, respectively) for these two NSC populations from embryogenesis to adulthood. They showed that the embryonic radial glia precursor parents of SGZ and SVZ NSCs are very similar. Over the first three postnatal weeks, both radial glia parent cells gradually shift to a quiescent adult NSC state. In this dormant state, genes responsible for controlling their niche environment are activated, whereas genes that sustain an active, proliferative and pro-differentiation state are transcriptionally shut off. Furthermore, both populations regain a development-like condition and express pro-neurogenic genes when reactivated to produce adult-born offspring. In fact, Borrett et al. (2022) analyzed the SGZ NSC data set of Hochgerner et al. (2018), looking for mRNAs that were increased during development from embryonic life to maturity but downregulated in activated adult intermediate progenitors (IPs). Of 105 SGZ NSC mRNAs that met these requirements, it is noteworthy that they found 94 mRNAs that were upregulated also in SVZ as they postnatally transitioned to quiescence and were then downregulated in the activated TAPCs/IPs (transit-amplifying progenitor cells/intermediate progenitors, i.e., type-2 and type-3 cells). These genes are involved in ion and neurotransmitter transport, cell-cell and cell-matrix interaction and lipid metabolism. They include for example, the sodium-potassium ATPase subunit *Atp1a2*, the inhibitor of cysteine proteases *Cst3*, the neural plasticity regulators neuroxexins (*Nrxn1/2*), and the genes involved in GABA neurotransmitter metabolism such as the two GABA transporter mRNAs *Slc6a11* and *Slc6a1* and the GABA-A receptor subunit mRNA *Gabrb1.* These genes are upregulated during embryogenesis and postnatally, maximally in dormant NSCs, while they are strongly downregulated during NSC activation, i.e., in TAPCs. These data are consistent with literature, showing that the activation of the GABA pathway maintains the quiescence of NSCs and *vice versa* its inhibition favors proliferation of progenitor cells (Song et al., 2012).

Conversely, three reports studied the NSC transcriptome during aging.

- Ibrayeva et al. (2021) investigated the process of mouse NSC quiescence during aging by performing single-cell clonal lineage tracing and transcriptomic analyses. They observed that the total number of NSCs decreases with age and that in parallel the percentage of NSCs in quiescence increases. Single-cell lineage tracing indicated the presence of different subpopulations of NSCs in the hippocampus. Importantly, authors find that Ascl1-labeled NSCs are a short-term subpopulation, as they undergo rapid clonal depletion due to commitment to neuronal differentiation, while Nestin-labeled NSCs are long-lived as they slowly divide to generate differentiated progeny and new NSCs. This subpopulation declines with age, but this is not due to accelerated differentiation: in fact, Nestin-labeled NSCs divide less frequently and symmetrically over time, with an increase of quiescent Nestin-labeled cells during aging. As a whole, in the adulthood and during aging, NSCs would replicate according to a gradual pattern of increased self-renewal that favors the conservation of the pool by reducing neurogenesis; this age-associated change consists in a shift from a population of NSCs dividing repetitively to a more quiescent population.
- Similar conclusions are reached by a study of Harris et al. (2021) showing that when mouse adult neurogenesis begins, all dividing NSCs are rapidly depleted through differentiation, while by 6 months of age more than half of the proliferating NSCs return to quiescence. Harris et al. (2021) distinguish between NSCs that return to quiescence (resting NSCs) and those that have never divided (dormant) and establish by RNA-seq and post-translational analyses that as age increases there is a decline of the pro-activation Ascl1 protein.
- To uncover the mechanisms of the age-dependent increase in quiescence of NSCs, Ibrayeva et al. (2021) performed single-cell RNA sequencing of neural stem/progenitor cells isolated from 2- and 4.5-month-old *Nestin/CFP* mice and identified NSCs in active or quiescent state using well-established markers. They found that in 4.5-month-old mice NSCs begin to enter deeper quiescence compared to 2-month-old mice and identified markers of molecular aging in the mature hippocampus, including the tyrosine-protein kinase Abl1. Other genes expressed during deeper NSC quiescence were involved in neurogenesis (*Sox2*, *Sox11*, *Wnt3 and Epha4*), in self-renewal (*Ezh2*, *Disc1*, *Mag*, *and Plp1*), and in cell cycle exit (*Wee1*, *Nsl1*, *Mcm6*, and *Heca*); furthermore, the authors observed changes in signaling regulators (*Abl1*, *Abl2*, *Crh*, and *Lef1*), Semaphorin signaling (*Plxna4*, *Plxnb3*, *Nrp2*, *and Farp2*), Ras signaling (*Arhger11*, *Arhgep32*, *Rassf1*, *Cdh13*, and *Icmt*), and Rho signaling (*Spata13*, *Myo9b*, *Tiam2*, *Mcf2l*, and *Scai*), known to regulate adult NSC quiescence or progenitor proliferation.
- Another study analyzed recently the process of neurogenesis and neuroinflammation during aging by single-cell RNA-seq of dentate gyrus combined with spatial transcriptomics, in young adult (3-month-old), middle-aged (9–11-month-old) and old (16–21-month-old) wild-type mice, from activation of quiescent NSCs until their maturation (Wu et al., 2025). Authors identified 11 cell populations of dentate gyrus resident cells, whose neurogenic lineages are quiescent NSCs, active neural progenitor cells (NPCs), neuroblasts and immature neurons. Authors identified a gene set (Core Aging Signature, CAS) composed of 95 upregulated and 248 downregulated genes, whose expression suggested that quiescent NSCs undergo early transcriptomic alterations already at middle age, indicating early molecular aging, in agreement with previous data (Harris et al., 2021; Ibrayeva et al., 2021). Notably, top enriched GO terms of CAS-Up were related to inflammation mediated by T-cells, which accumulated in aged dentate gyrus and displayed upregulation of IFN-gamma. Then, authors tested whether neuroinflammation during aging could be due to blood brain barrier (BBB) deficiency by using the Pdgfb^ret/ret^ mouse, which indeed showed pericyte decrease associated to infiltration of T cells and reduced number of neuroblasts and immature neurons (DCX-positive) in the dentate gyrus. Conversely, no change occurred for Sox2-positive NSC/progenitor cell. This may indicate the existence of additional age-dependent mechanisms or also that NSC, by remaining quiescent, are less vulnerable to T cell signaling.

See Table 2 for section summary.

**TABLE 2:** Mouse hippocampal neurogenesis - single-cell/bulk RNA sequencing from isolated dentate gyrus.

Aim	Conclusions/key findings	Genes/cell types	Transcriptomic analysis details	Marker usage/Methods	References
Defining molecular dynamics of DG cell types in neurogenesis, of perinatal, juvenile, and adult mice	Conserved gene regulation in perinatal and adult neurogenesis. Proposed model: cell type defined transitions rather than a continuous process, with long-lived self-renewing RGL cells, predominantly quiescent, even during early post-natal development	Top enriched markers in RGL cells: *Id3*, *Sox9*, *Hes1*, *Tshz2*, *Hopx*, *Hes5*, *Tfap2c*, *Cox4l2*, *Rgs5*, *Rhcg*, and *Ascl1*; absence of expression of cell cycle genes (*Cdk1* and *Top2a*) indicates that these NSCs are mainly quiescent	scRNA-seq of DG at E16.5 and between P0-P132	Standard markers to identify cell types. NSCs identified for their similarity to astrocytes (*Gfap, Hes5, and Sox9*) and expression of *Lpar1*	Hochgerner et al. (2018)
Characterization of DG precursor population labeled by Hopx	Proposed model: DG neurogenesis as a continuous process. A common neural progenitor population (Hopx+) contributes to neurogenesis throughout development from embryonic stage to adulthood giving rise to granule neurons and adult RGLs	From embryonic to adult stage, Hopx + progenitors generate granule neurons and show increased expression of cell cycle genes (*Cdk1*, *Prc1*), chromatin modification genes (*Smarcad1*, *Trim28*), cell signaling (*Nrxn1*, *Serpine2*), lipid metabolism (*ApoE*)	scRNA-seq on developing hippocampus (E15.5 and P4) or P45 DG	Isolation by FACS of Hopx + neural progenitors of *Hopx-CreER::H2B-GFP* mouse	Berg et al (2019)
Study of DCX-positive immature neurons	Immature cells are enriched in genes associated to neurodegeneration while more mature cells are enriched in autism-related genes	Identification of the gene *PRR5L* as marker of imGCs. Hopx is maximally expressed in NSCs	scRNA-seq on DG from adult (P51-P66) mice	Isolation by FACS of DG cells from adult transgenic *Dcx-DsRed* mice	Gao et al. (2017)
Investigation of the molecular cascade underlying NSC development in early post-natal mouse DG	Identified two sequential steps in the transition of NSCs to quiescence, with an autophagy burst before NSC quiescence acquisition and increase of cellular reactive oxygen species along NSC maturation	Downregulation of *Nfix*, *Top2a*, *Rps3* and *Sox11*, and upregulation of *Npas3*, *Hopx* and *Sox9* during transition of NSCs to an early quiescent state	scRNA-seq on DG from P3, P7 and P14 mice	Isolation by FACS of Hopx + neural progenitors of the DG dissected from *Hopx3FlagGFP/+* mouse	Jimenez-Cyrus et al. (2024)
Defining the gene signatures of quiescent NSCs and their early progeny in the adult mouse hippocampus	Activation of quiescent NSCs is accompanied by downregulation of Notch signaling, GABA and BMP pathways, fatty acid oxidation, sphingolipid and glutathione metabolism, according to a shift of metabolism during activation	Identified 41 transcription factors downregulated and 42 upregulated during hippocampal neurogenesis, including genes known to participate to neurogenesis, as *Sox2*, *Sox9*, *Id3*, *p53*, and other genes such as *Dbx2*, *Id4*, or *N-myc*	scRNA-seq analysis on adult mouse DG	Isolation by FACS of neural precursors from transgenic mice *Nestin-CFP* ^*nuc*^. Developmental trajectories at single-cell resolution were reconstructed using the Waterfall pipeline	Shin et al. (2015)
Comparison of transcriptional trajectory of SGZ and V-SVZ NSCs during development (from embryo to maturity)	Radial glia parent cells gradually shift to a quiescent adult NSC state during the first three post-natal weeks. In this dormant state, genes that sustain a proliferative state are downregulated. NSCs in the two niches share common trajectories	Genes upregulated during embryogenesis and postnatally, maximally in dormant NSCs, and downregulated during adult NSC activation include the Na/K ATPase subunit *Atp1a2*, the inhibitor of cysteine proteases *Cst3*, the GABA transporter mRNAs *Slc6a11* and *Slc6a1* and the GABA-A receptor subunit *Gabrb1*	scRNA-seq of mouse DG from E16.5 to P132 by Hochgerner et al. (2018); scRNA-seq of V-SVZ from E14 to P6 by Borrett et al. (2020)	--	Borrett et al. (2022)
Study of the process of NSC quiescence during aging	NSCs subpopulations show asynchronous decline during aging. Aging is associated with a shift from a subpopulation of NSCs dividing repeatedly (Ascl1-labeled NSCs) to a more quiescent subpopulation (nestin-labeled NSCs)	Identification of tyrosine-protein kinase *Abl1* as NSC pro-aging factor. Other DE genes during deep quiescence are involved in neurogenesis (*Sox2*, *Sox11*, *Wnt3*, and *Epha4*) in self-renewal (*Ezh2*, *Disc1*, *Mag*, and *Plp1*), and in cell cycle exit (*Wee1*, *Nsl1*, *Mcm6*, and *Heca*)	scRNA seq on DG from 2- and 4.5-month-old mice	Isolation by FACS of neural stem/progenitors from *Nestin/CFP* mice and identification of NSCs in active or quiescent states using specific markers	Ibrayeva et al. (2021)
Investigation of molecular mechanisms underlying lifelong maintenance of hippocampal NSCs	During aging, more of dividing NSCs return to quiescence, instead of differentiating (resting cells). These NSCs are distinct from quiescent cells that have never proliferated (dormant NSCs)	As age increases there is degradation of the pro-activation Ascl1 protein by HUWE1	scRNA-seq on DG from 1, 2 and 6–8 month-old mice	RNA-seq performed on NSCs isolated by FACS from *Ki67* ^*TD*^ */Nestin-GFP* mice. Tet off-H2B-GFP mice used to study NSCs divisions	Harris et al. (2021)
Study of neurogenesis and neuroinflammation during aging, from activation of quiescent NSCs until their maturation	Top enriched GO terms of Core Aging Signature (CAS) upregulated genes were related to inflammation mediated by T-cells. The *Pdgfb* ^ret/ret^ mouse with BBB deficit showed reduced number of DG neuroblasts and normal number of NSCs, indicating different cell regulations	Identification of a gene set (CAS) composed of 95 genes upregulated and 248 genes downregulated, whose expression suggested that quiescent NSCs had early transcriptomic alterations already at middle age	Spatial scRNA-seq on DG from young adult (3-month-old), middle-aged (9–11-month-old) and old (16–21-month-old) wild-type mice	Wild-type mice used for DG scRNA-seq studies. *Pdgfb* ^ret/ret^ mouse with BBB deficit used to test whether this deficit caused neuroinflammation during aging	Wu et al. (2025)
Developmental transcriptional profile of nestin-GFP-expressing stem/progenitor cells	Identified cell-autonomous factors that regulate neural cell progenitor development	Identified 9 genes, 3 downregulated (*CD47* and *Cspg2* involved in cell adhesion; *Tbx5* transcriptional regulator); 6 upregulated (*ApoE*, lipid metabolism regulator; *Cldn10* and *Vtn* cell adhesion; *Eaat2/GltI*, glutamate transporter) in P28 progenitors vs. P7 controls	Microarray analysis on DG from P7 and P28 mice	Isolation by FACS of stem/progenitor cells from *Nestin-GFP* transgenic mice	Gilley et al. (2011)

Abbreviations: BBB, blood-brain barrier; DG, dentate gyrus; E, embryonic day; GC, granule cells; ImGC, immature GC; NSC, neural stem cell; P, postnatal day; RGL, radial glial like; sc/snRNA-seq, single-cell/single-nucleus RNA, sequencing; V-SVZ, ventricular-subventricular zone.

### Mouse dentate gyrus neurogenesis–transcriptome analysis following a neurogenic stimulus

Here we will review studies in which the transcriptional profile of the dentate gyrus was analyzed after mice were exposed to the neurogenic stimulus of voluntary running, with a focus on stem/progenitor cells. It is worth noting that neurogenic stimuli (such as running, antidepressants, enriched environment and dietary components) can promote proliferation of NPCs in the dentate gyrus but not of quiescent NSCs (Kronenberg et al., 2003; Ceccarelli et al., 2020), as specific mechanisms maintain their quiescent state.

Indeed, a study by Micheli et al. (2023) performed on the isolated dentate gyrus of aged mice revealed the role of the cell cycle inhibitor p16Ink4a in maintaining NSCs quiescence in aged mice, by preventing NSC activation after the stimulus of running. In fact, if *p16Ink4a* was deleted, quiescent NSCs were strongly activated by 12 days of running. In particular, by RNA-seq of *p16Ink4a* knockout mice dentate gyrus and comparative analyses through the DESeq2 software, the authors identified 106 genes whose differential expression specifically reflects the pattern of proliferative response of *p16Ink4a* knockout NSCs to running. Upregulated genes include: *i)* the activator of hippocampal neurogenesis *Tfap2c* (Mateus-Pinheiro et al., 2017); *ii)* the negative regulators of ROS levels *Nlrc5*, *Gstm2* and *Mocos*, whose upregulation is consistent with the decrease of ROS levels occurring at NSC activation (Adusumilli et al., 2021); *iii)* the regulator of fatty acid synthesis *Lpin2*; *iv)* the regulator of glucose metabolism *Insm2*. Moreover, cell cycle regulators such as *Top2a*, *Prc1*, and *Pole* were upregulated, being implicated in DNA replication and cytokinesis and thus possibly involved in the proliferative activation of p16 knockout NSCs by running. Furthermore, Micheli et al. (2023) demonstrated that the regulation of these genes correlates with the NSC reactivity to repeated neurogenic stimuli, thus supporting the idea that they play a role as regulators of NSC activation. Interestingly, none of the 106 genes related to activation of NSCs identified by Micheli et al. (2023) overlaps with the about 70 most representative genes differentially expressed in aging quiescent NSCs (Ibrayeva et al., 2021), which is consistent with the different status of cells analyzed (activated or quiescent cells, respectively).

In a similar study using another mouse model - i.e., the knockout of the cell cycle inhibitor *Btg1* - where NSCs are responsive to a neurogenic stimulus, Micheli et al. (2021) sought to identify genes differentially expressed during the activation of dentate gyrus NSCs induced by voluntary running. Sedentary Btg1 knockout mice present a severe depletion of the NSC pool, with elevated levels of apoptosis, thus configuring a condition of premature neural aging. By RNA-seq of isolated dentate gyri, the authors identified 42 genes upregulated and 42 downregulated in Btg1 knockout compared to wild-type mice in basal condition that were counter-regulated by running. Among the 42 down- and counter-regulated genes, alpha-synuclein (*Snca*), *Fos*, *Arc*, and *Npas4* showed significantly greater differential regulation (for *Snca* log2 fold change was about 20). These genes are implicated in aging and regulate neuronal proliferation, apoptosis, plasticity, and memory. In particular, the authors showed that restoring the *Snca* levels in the dentate gyrus of Btg1 knockout mice reverses the aging phenotype, i.e., the defective neurogenesis, and concluded that the above genes could exert a positive regulatory action on NSC maintenance and play a functional role in the process of brain aging present in this model.

Another report, by Walker et al. (2016) showed that type-1 NSCs and type-2a precursor cells in the adult mouse dentate gyrus express lysophosphatidic acid receptor 1 (*Lpa1*), and that increased expression of Lpa1 is associated with the induction of neural proliferation induced by running. Furthermore, the authors demonstrated that infusion of LPA in the hippocampus stimulates the proliferation of progenitor cells. They also performed transcriptome analysis by RNA sequencing of Lpa1-GFP-expressing neurospheres obtained from hippocampal precursor cells sorted by FACS. In this way they showed that Lpa1 signals via the AKT and MAPK pathways, thus clarifying its functional context. See details in the section “Effect of the deregulation of specific genes on dentate gyrus neurogenesis and transcription”.

It is worth mentioning three papers, reporting transcriptomic analyses of dentate gyrus RNA after adult neurogenesis was induced by running, though no attention was given to NSC role (Grégoire et al., 2018; Guerrieri and van Praag, 2015; Chatzi et al., 2019).

Grégoire et al. (2018) performed RNA-seq analysis of the dentate gyrus from mice subjected to three different protocols of running (more or less severe, or plus social enrichment) and distinguished between runners (RUN; low or high runners: L-RUN, H-RUN). 178 genes were found to be significantly changed in the RUN group and enriched in gene sets associated with glutamatergic synapses, phosphatidylinositol and calcium signaling, circadian entrainment, LTP, and cGMP-pKG signaling.

Guerrieri and van Praag (2015) stimulated adult neurogenesis in mice either by running or by administering an AMP-Kinase agonist for 3, 7 or 14 days, and they found that both treatments can similarly stimulate neurogenesis after 7 days, as detected by measuring BrdU-positive cells in the SGZ of the dentate gyrus. By performing microarray analysis authors found 192 genes upregulated and 142 downregulated in common to both treatments for 7 days; these genes are mostly involved in neuronal plasticity, e.g., *Grit* (downregulated) that inhibits BDNF-induced axonal branching, or *Hap1* that regulates postnatal neurogenesis through mTorc1. However, in the longer period (14 days of treatment) only running was able to stimulate neurogenesis.

Chatzi et al. (2019) studied synaptic changes elicited by running in the dentate gyrus, by monitoring neuron activation through c-Fos expression using conditional Fos-TRAP mice where the activation of c-Fos is permanent once occurred. By RNA-seq they identified *Mtss1L*, whose knockdown *in vivo* prevented the exercise-induced increases in spines and excitatory postsynaptic currents.

See Table 3 for summary of the above discussed studies.

**TABLE 3:** Mouse dentate gyrus neurogenesis–transcriptome analysis following a neurogenic stimulus.

Aim	Genes regulated/cell types and key findings	Stimulus/Model	Marker usage/Methods	References
Identification of genes associated with NSC activation by running in the DG of aged *p16Ink4a*-KO mice	Identified 106 genes correlated with NSC activation by running in DG of *p16Ink4a*-KO mice. Upregulated genes: negative regulators of ROS levels, metabolic and cell cycle regulators	RNA-seq of DG from *p16Ink4a*-KO (12-month-old) mice subjected to running (12 days)	Comparative analyses of data from DESeq2 software	Micheli et al. (2023)
Search for genes involved in DG NSCs activation by running, in a model of premature neural aging, *Btg1*- KO mice	Identification of *Snca*, and possibly other 83 genes implicated in maintaining NSCs pool and preventing DG aging in this model. *Snca* overexpression in DG restores defective neurogenesis	RNA-seq of isolated DG from adult *Btg1-*KO mice subjected to voluntary running (12 days)	Activated NSCs genes were identified by comparative analyses of data from Cuffdiff software	Micheli et al. (2021)
Study of the role of lysophosphatidic acid receptor 1 (Lpa1) in NSC of DG	The expression of Lpa1 is associated to the induction of neural proliferation by running. Lpa1 acts via the AKT and MAPK pathways	RNA-seq of neurospheres obtained from hippocampal Lpa1-GFP precursor cells. Running (10 days)	Standard markers were used for DG immunohistochemistry	Walker et al. (2016)
Identification of gene signature of DG from mice subjected to three different protocols of running	178 genes were found to be significantly changed by running. RUN-induced genes are enriched in data sets associated with glutamatergic synapses, phosphatidylinositol and calcium signaling, circadian entrainment, LTP, and cGMP-pKG signaling	RNA-seq of whole DG micro-dissected from adult CD1 mice Running -High Runner (4 weeks) -Low-Runner (4 weeks) -Running-independent complex environment	Standard markers were used for DG immunohistochemistry	Grégoire et al. (2018)
Analysis of the stimulation of adult neurogenesis in mice by either running or by administering an AMP-Kinase agonist (AICAR)	Identified 192 genes upregulated and 142 downregulated in common to both treatments for 7 days, involved in neuronal plasticity	Microarray analysis of isolated DG (1-month-old C57BL/6J) Running or AICAR treatment: 3- 7–14 days	Standard markers were used for DG immunohistochemistry	Guerrieri and van Praag (2015)
Study of synaptic changes elicited by running in the DG	Identification of *Mtss1L*, as a novel effector of stimulus-dependent rearrangement of synapses. Mtss1L knockdown *in vivo* prevents the exercise-induced increases in spines and excitatory postsynaptic currents	RNA-seq of laser capture micro-dissected DG granule cells from *cFos* ^*creERT2*^ transgenic mice (Fos-TRAP), 3 and 7 days after 2 h of voluntary exercise	*Fos-TRAP* mice are used to monitor neuron activation	Chatzi et al. (2019)

Abbreviations: DG, dentate gyrus; NSC, neural stem cell; RGL, radial glial like.

Moreover, it is known that NSC pool is activated in pathological conditions, such as in a mouse model of mesial temporal lobe epilepsy (MTLE) induced by intracerebral injection of Kainic acid (KA) or after traumatic brain injury (TBI). In these conditions, characterized by neuronal hyperactivity, the NSCs become reactive NSCs (react-NSCs), which differentiate into reactive astrocytes, a cellular type linked to astrogliosis. This process was well described in the paper of Abiega et al. (2024), where the authors compared NSCs and react-NSCs at 3dp MTLE induction by RNA-seq.

### Mouse dentate gyrus neurogenesis–comparison of gene profiles

In order to highlight which genes are most representative of the quiescent or activated state of NSC and progenitor cells, we performed pairwise comparisons between the representative genes regulated during NSC activation or quiescence revealed by the mouse transcriptomic studies cited in the previous sections.

If we compare the 70 genes associated with NSC deeper quiescence during aging identified by Ibrayeva et al. (2021) with the 83 transcription factors involved in NSC activation identified by Shin et al. (2015) (Figure 4A inside the reference), we find 3 genes in common (*Mycn*, *Lef1*, *Fos*). In contrast, no overlaps are found between the 70 genes highlighted by Ibrayeva et al. (2021) and those highlighted by Borrett et al. (2022) (Table 6 inside the reference; 105 genes selectively increased in quiescent adult NSC), Berg et al. (2019) (5 NSC-specific genes shown in Figure 4D inside the reference), Hochgerner et al. (2018) (Figure 2C inside the reference; 11 top enriched genes in RGL during early postnatal neurogenesis), or Jimenez-Cyrus et al. (2024) (Figure 3B inside the reference; 34 genes up- or downregulated specifically during postnatal transition of active NSCs to early quiescence). This suggests that the genes regulated during the aging-induced quiescence identified by Ibrayeva et al. (2021) involve processes that differ at least partially from those of progressive NSC activation and/or quiescence pinpointed in the other studies.

Conversely, the Jimenez-Cyrus et al. (2024) gene set, involved in the postnatal transition to early quiescence of NSCs, has 14 genes in common with the 83 transcription factors identified by Shin et al. (2015), 2 genes with the NSC-specific genes identified by Berg et al. (2019) and 2 genes with those upregulated by running in the dentate gyrus of Btg1 or p16 knockout mice (Micheli et al., 2021; 2023).

Interestingly, among the 11 genes top-enriched in RGLs identified by Hochgerner et al. (2018), 4 genes (*Sox9*, *Id3*, *Hes1*, and *Hopx*) are in common with the transcription factors identified by Shin et al. (2015), 2 genes (*Sox9* and *Hes1*) are in common with the NSC-specific genes highlighted by Berg et al. (2019), 1 gene (Sox9) with genes identified by Borrett et al. (2022) and 1 gene is in common with genes identified by Micheli et al. (2023), namely, *Tfap2c*, the top gene induced after NSC activation by running in p16 knockout dentate gyrus. Hochgerner et al. (2018) show that *Tfap2c* is expressed according to a gradient, low in quiescent RGL and maximal in progenitor cells, in parallel to the cell cycle gene *Cdk1*.

Furthermore, among the top 150 up- and downregulated genes included in the UP^1000^ and DOWN^1000^ genes regulated during neurogenesis identified in the study on quiescent and activated NSCs by Shin et al. (2015) (Figure 4B inside the reference), 58 genes overlap with the genes enriched in quiescent NSCs identified by Borrett et al. (2022) (Table 6 inside the reference), whereas 8 genes overlap with the 106 top regulated genes during NSC activation by running in p16Ink4a knockout dentate gyrus (Micheli et al., 2023; Supplementary Table S6), namely, the proliferation genes *Top2a*, *Prc1*, *Ki67*, *Knstrn*, the radial glia regulator *Eomes*, *Nrxn1*, *Rps5*, and *Rps12*.

Furthermore, among the genes identified in quiescent NSCs by Borrett et al. (2022), *Sox9* is in common with the NSC genes highlighted by Berg et al. (2019) and *Nrxn1* is in common with genes identified in activated NSCs by Micheli et al. (2023), while *ApoE* is in common with genes differentially expressed in dentate gyrus during postnatal development (P28 vs. P7), identified through microarray analysis of Nestin-GFP-expressing stem/progenitor cells (Gilley et al., 2011; Table 1 inside the reference; 8 genes).

These overlaps may help to define sets of genes that have been consistently found to be regulated during activation and during the following quiescence of NSCs in multiple studies.

Moreover, it is worth noting a convergence of transcriptome results from different studies focused on the complex lipid metabolism regulation during NSC activation, namely, the studies: a) by Shin et al. (2015) demonstrating that a decrease of fatty acid oxidation is associated with activation of NSCs in the dentate gyrus; b) by Knobloch et al. (2013), see below) showing that *de novo* FASN-dependent lipogenesis is required for neural stem/progenitor cells neurogenesis activation (while an increase of the breakdown of fatty acids, i.e., oxidation, leads to quiescence of NSCs (Knobloch et al., 2017)); and c) by Micheli et al. (2023) showing that *Lpin2*, a key enzyme involved in the generation of fatty acids through the production of triacylglycerols, is upregulated in activated NSCs.

See all gene overlaps in Supplementary Table S1.

### Effect of the deregulation of specific genes on dentate gyrus neurogenesis and transcription

The papers discussed so far highlight that the NSCs and NPCs are heterogeneous populations that follow a progressive sequence of molecular and cellular changes. Understanding their temporal development is crucial for advancing knowledge about adult neurogenesis.

In this section, we provide a comprehensive summary of studies examining the effects of gene deletion or overexpression on the regulation of adult neurogenesis. These include genes with well-established roles in neurogenesis and others whose regulatory function in this process is less understood. Specifically, we have selected studies focusing on the gene expression profiles in the NSCs/NPCs of the dentate gyrus, often combined with lineage tracing approaches, emphasizing factors identified through transcriptomic studies discussed in the previous sections of this review. The genes are categorized based on the cellular populations in which they are expressed or the process they regulate.

See Supplementary Table S2 for summary.

- NSC stemness/self-renewal

*EED* - Liu et al. (2019) sought to identify the function in neurogenesis of EED (Embryonic Ectoderm Development), core component of the Polycomb complex. They showed that *EED* deletion in mouse neural stem/progenitor cells by conditional knockout of *EED* driven by hGFAP-Cre inhibited neurogenesis *in vivo*, with a strong decrease of neuroblasts and immature neurons (DCX^+^ cells), as well as *in vitro*, with reduced proliferation of primary neural stem/progenitor cells in neurosphere cultures. By averaging RNA-seq results from two biological replicates from either *EED* knockout or wild-type P14 dentate gyrus, Liu et al. identified 561 upregulated genes and 486 downregulated genes, involved in forebrain development and neuronal differentiation. Further analysis by category netplot identified *Prox1* and *Sox11* as best candidate genes that are downregulated by EED during neurogenesis, while the cell cycle kinase inhibitor *p16Ink4a* (*Cdkn2a*) was one of the most upregulated genes. Interestingly, they showed that overexpression of *Sox11*, or downregulation of p16Ink4a using a lentivirus expressing *Cdkn2a*-shRNA, can reverse the proliferation defect of EED-knockout neural stem/progenitor cells cultured *in vitro.* This finding agrees with the negative key regulatory role played by p16Ink4a on dentate gyrus neurogenesis, as shown by Micheli et al. (2019). The authors conclude that EED is required for NSC/progenitor cells self-renewal in the DG.

*LPA1* - Walker et al. (2016) showed that self-renewing NSCs and type-2a progenitor cells in adult dentate gyrus express the lysophosphatidic acid receptor 1 (LPA1). LPA1 was detected using the *LPA1-GFP* reporter mouse line. By FACS-sorting the authors identified two distinct populations: proliferative cells (LPA1-GFP^+^/EGFR^+^/prominin-1^+^) and quiescent cells (LPA1-GFP^+^/EGFR^−^/prominin-1^-^). Transcriptional profiling of isolated LPA1-GFP^+^/EGFR^+^ proliferative precursor cells suggested immune-like characteristics with the chemokine signaling pathway identified as regulator of adult hippocampal precursor cell proliferation. Consistently, Walker et al. observed that CXCL1 treatment increased the number of neurospheres generated from both the SVZ and the dentate gyrus. Comparison of a list of 145 genes enriched in proliferative precursor cells with a previously generated transcriptomic profile of proliferating stem/precursor cells expressing Sox2 (Bracko et al., 2012), revealed that only three genes (*Igf1*, *Dab2*, and *Txnip*) overlapped, suggesting that the two populations of LPA1-GFP^+^/EGFR^+^ and Sox2^+^ cells only partially overlap; we have to remember that Sox2 specifically labels activated NSCs (type-1) and type-2a progenitor cells (Steiner et al., 2006). LPA1 appears to have an intrinsic ability to promote adult hippocampal neurogenesis by enhancing cell survival through activation of AKT and MAPK pathway, without, however, affecting proliferation. In conclusion, the transcriptomic profile of LPA1^+^ proliferating NSCs in dentate gyrus includes genes involved in the cytokine signaling, suggesting a cross talk between NSCs and immune system.

*FoxO3* - Schmidt-Strassburger et al. (2012) investigated the gene pathways activated by the *FoxO3* gene in the mouse dentate gyrus and in forebrain, and responsible of *FoxO3* requirement for NSC self-renewal, as previously shown (Renault et al., 2009). Overexpressing *FoxO3* in CamKII-positive cells of the forebrain and in neuroblasts and neurons of the dentate gyrus by a conditional tetracycline-off transgenic mouse (Arruda-Carvalho et al., 2014), they observed a decrease of brain size, including the dentate gyrus, and an increase of apoptosis in the dentate gyrus of 3-month-old animals but not in the cortex or striatum. Through RNA microarray of the forebrains of *FoxO3* transgenic mice, the authors identified *Pik3ip1* as a target gene of FoxO3, which can enhance pre-existing pro-apoptotic stimuli. Moreover, they suggested that FoxO3 is necessary for NSC self-renewal by regulating oxygen metabolism via induction of the gene encoding hypoxia-inducible factor 1 (*HIF1*), identified by microarray, which promotes self-renewal of NSCs by improving oxygen availability to NSCs, located in close proximity to blood vessels *in vivo*.

*Mbd1* - Jobe et al. (2017) demonstrated that methyl-CpG-binding domain 1 (Mbd1), a DNA methylation “reader,” is required for the proper differentiation of adult dentate gyrus stem cells into mature neurons. In fact, the authors found that in the dentate gyrus of *Mbd1* knockout mice there is a decrease of immature neurons and an increase of double-labeled GFAP and betaIII-tubulin-positive cells, as well as of type-2a/b cells, indicating a defect in NSC maturation. This was confirmed by RNA-seq of Nestin^+^ cells isolated by FACS from adult *Mbd1* knockout dentate gyrus, showing an enrichment of upregulated astrocyte-lineage genes, such as *Atp13a4*, *Cd38*, *Chrdl1*, *Gjb6*, *Gli2*, *Gm973*, *Gpr179*, *Grin2c*, *Hsd11b1*, *Rhcg*, *Slc39a12*, and a downregulation of neuronal genes. Thus, Mbd1 appears to be important for the neurogenic potency and the integrity of dentate gyrus NSCs.

Sirt1 - Ma et al. (2014) investigated the role of Sirt1 in the control of adult mouse hippocampal neurogenesis. They showed that *Sirt1* is expressed in NSCs as well as in amplifying progenitor cells and immature neurons of the dentate gyrus. Selective knockout of *Sirt1* in stem and progenitor cells expressing Nestin led to an increase in stem and progenitor cell proliferation, without affecting differentiation. Similar effects were observed in *Sirt1* knockout neurospheres. The authors conclude that Sirt1 negatively regulates the self-renewal process of stem and progenitor cells. A microarray profiling of *Sirt1* knockout, or wild-type neurospheres treated with the Sirt1 agonist resveratrol, showed that genes regulated by *Sirt1* knockout and counter-regulated by resveratrol included genes involved in metabolic pathways, such as hexokinase 3 (*HK3*), malate dehydrogenase 1 (*Mdh1*) and nicotinamide N-methyltransferase (*Nmt*), as well as genes involved in neurogenesis including *EphA4*, *Sox7*, *Sox10*, *Fgfr3*, and *Erbb4*. Moreover, *Sirt1* knockout mice exhibited increased expression of mediators of Notch signaling, such as *Dll4* and *Hes5*, while their expression decreased after resveratrol treatment. This suggests that Sirt1 paradoxically inhibits the pro-quiescence Notch signaling in stem and progenitor cells.

The authors conclude that Sirt1 decreases NSC self-renewal prior to neuronal fate commitment. Reduced Sirt1 level/activity results in increased expression of “stemness” genes (i.e., NSC activatory genes) and enhanced self-renewal. Thus, Sirt1 appears to function as an endogenous negative regulator of “stemness” activating genes.

*Sox2* - Bracko et al. (2012) analyzed by whole-genome microarray the gene expression signatures of mouse dentate gyrus NSCs, identified as Sox2-positive cells, and of progenitor cells/immature neurons, identified as DCX-positive cells. These cells were isolated from transgenic mice expressing GFP under the *Sox2* promoter and DsRed under the *DCX* promoter, respectively. Authors compared the transcriptional profile of isolated dentate gyrus cells and observed that 3.4% of all transcripts analyzed were differentially regulated between Sox2^+^ and DCX^+^ cells. Sox2^+^ cells were enriched in GO terms of genes involved in developmental processes and cell differentiation as well as energy generation and lipid metabolism (including *Bmp6*, *ApoE*, *Acacb*, *Igfbp7*, *Edf1*, *Irs*, *Lrp1*, *Cpt1a*, *Pparc1a*, *Acaa2*, and *Acsl6*). In contrast, DCX^+^ cells were enriched in GO terms of genes associated with neurogenesis and neuron generation. The most highly expressed gene in Sox2-positive cells compared to DCX-positive cells was *Igf2*. Interestingly, Bracko et al. demonstrated that Igf2 is not only highly expressed in NSCs (Sox2^+^) but also that it stimulates proliferation of cultured dentate gyrus NSCs, as judged by short hairpin RNA-mediated *Igf2* knockdown, acting via AKT-dependent signaling. Thus, AKT appears to integrate pro-proliferative signals, such as Igf2, in opposition to pathways inducing quiescence and differentiation, such as BMP (Mathieu et al., 2008; Farioli-Vecchioli et al., 2014).

- NSC quiescence

*Sox9* - *Sox9* was found to be enriched in RGL cells and during transition of NSCs to an early quiescent state (Hochgerner et al., 2018; Jimenez-Cyrus et al., 2024). Caramello et al. (2022) showed that the conditional deletion of *Sox9* in the mouse archicortex, which gives rise to the primitive dentate gyrus (dentate neuroepithelium), causes downregulation of the NSC marker *Hopx*. This was accompanied by a decrease of NSCs formation, suggesting that Sox9 expression, together with Hopx, triggers NSCs development in the adult dentate gyrus. The authors identified *Hopx* as a *Sox9*-regulated gene in the cortical hem (CH, embryonic organizer of the hippocampus, part of the archicortex) by comparing the transcriptome of the archicortex fully and partially deleted for *Sox9*. However, since Sox9 and Hopx are highly expressed in CH cells, which do not possess NSC properties, the authors concluded that Sox9 and Hopx contribute to astrocytic differentiation rather than to NSC differentiation. This conclusion contrasts somewhat with that of Berg et al. (2019), who, based on the observation that Hopx labels RGL cells in SGZ since their embryonic stage, and then give rise to granule neurons, argues for a role of Hopx in NSC maintenance and neuron generation.

*REST* - Mukherjee et al. (2016) investigated the role of *REST* (repressor element 1-silencing transcription factor) in the mechanisms controlling self-renewal of adult NSC. Specific deletion of *REST* in NSCs, obtained by injecting a *GFAP-Cre-p2A mCherry* expressing retrovirus into the dentate gyrus of 6– to 8-week-old *REST flox/flox (fL/fL)* mice led to an increase of proliferating cells (Ki67-positive), as well as of neuroblasts and immature neurons (DCX-positive) and mature neurons (NeuN-positive). This suggests that REST maintains the quiescence of stem/progenitor cells and prevents their premature differentiation into adult-born neurons. By RNA seq of cultured mouse hippocampal NSCs electroporated with a *REST* shRNA vector to knockdown *REST*, compared with a control empty shRNA vector, authors found a gene expression profile indicative of increased differentiation in *REST* knockout progenitor cells. Furthermore, with a superimposed Chip-seq approach, they identified several genes unique to Ki67^+^ cells in the *REST* knockout group that are related to cell cycle and DNA replication and are overexpressed (e.g., *Cdc20*, *Cdk5r2*, *Tipin*, *Mms22l*, *Nom1*, *Rpl4*). In conclusion, the authors suggest that *REST* function is implicated in maintaining the quiescence of NSCs as well as the proliferative state of transit-amplifying progenitors to prevent premature differentiation.

*TGF-β1* - Previous work showed that intracerebroventricular injection of TGF-β1 in the adult rat brain suppressed neural progenitor cell proliferation (Wachs et al., 2006). Similarly, overexpression in transgenic mice of TGF-β1 in GFAP^+^cells inhibited brain progenitor cell proliferation (Buckwalter et al., 2006). Then, Kandasamy et al. (2014) generated a transgenic mouse that conditionally expresses *TGF-β1* in the hippocampal NSC niche under the Ca-Calmodulin kinase promoter, and showed that TGF-β1 promotes NSC quiescence and, concomitantly, neuronal survival. By microarray analysis of TGF-β1-treated hippocampal neurospheres the authors observed activation of the Smad pathway and regulation (prevalently downregulation) of several genes associated to cell cycle and cell proliferation, including *cyclin G1*, *E*, *D2* and *B1*, the cyclin-dependent kinase inhibitor 1C (*p57*), and the cyclin division cycle 20 homolog (*cdc20*). Furthermore, TGF-β1 induced neurogenesis-associated genes such as the bHLH transcription factor acheate-scute complex homolog-like 1 (*Mash1*), the Notch pathway gene *HES1*, the Notch ligands delta-like 1 (*dll1*) and jagged1 (*Jag1*), the stem and progenitor cells marker *Nestin*. This is consistent with previous findings that Notch1 and Notch2 maintain NSC quiescence in the dentate gyrus (Ables et al., 2010; Zhang et al., 2019). Given the high levels of TGF-β1 in ageing and neurodegenerative diseases, this molecule represents a potential target for future therapies. In particular TGF- β1 has been shown to exert a neuroprotective effect against fibrillary tangles in Alzheimer’s disease (Tominaga and Suzuki, 2019). The authors concluded that TGF-β1 promotes stem cell quiescence as well as generation of new neurons.

- NSC and NPC proliferation/activation

*Arid1a*–Arid1a (AT-rich interactive domain-containing protein 1A), a component of the SWI/SNF chromatin-remodeling complex, is required for SWI/SNF targeting and nucleosome remodeling and its absence causes aberrant gene expression. Liu et al. (2023) demonstrated that *Arid1a* gene knockout in cortex and hippocampus reduces neural stem/progenitor cell proliferation and differentiation into neurons (DCX^+^ neuroblasts and immature neurons) in the dentate gyrus, increasing perinatal and postnatal apoptosis and causing reduced hippocampal size. Furthermore, single-cell RNA-seq of hippocampal cells revealed that the *Prox1* gene (expressed in type-2b progenitor cells, neuroblasts and neurons) was significantly downregulated in *Arid1a* knockout mice. Notably, overexpression of *Prox1* in hippocampal cell cultures rescued the defect of stem/progenitor cell proliferation and differentiation caused by Arid1a deletion in hippocampus. The authors propose that Arid1a promotes the establishment and proliferation of NSC/progenitor cells in the dentate gyrus.

*Fasn* - Knobloch et al. (2013) investigated the role of cell metabolism in regulating the proliferation of mouse dentate gyrus neural stem and progenitor cells, focusing on the role of *de novo* lipogenesis controlled by the key enzyme Fasn (fatty acid synthase). They found that *Fasn* is highly expressed in proliferating neural stem and progenitor cells, in the subgranular zone (SGZ) of the dentate gyrus and in the SVZ. Furthermore, genetic or pharmacological inactivation of Fasn strongly reduced the proliferation of neural stem and progenitor cells, leading to a large loss of newly generated neurons and apoptosis. The authors further showed that the enzyme Spot14 decreases the availability of malonyl-CoA, a Fasn substrate for lipogenesis, and that Spot14- NSCs isolated by cell sorting from a knockout *Spot14-CreERT2* mouse present higher proliferation than Spot14^+^ cells. Gene expression analysis using Affymetrix GeneChip arrays showed enriched GO terms such as cell cycle and DNA replication in the upregulated genes of Spot14-cells. The authors conclude that Fasn-dependent *de novo* lipogenesis is required for activation of neural stem/progenitor cells neurogenesis.

lncRNAs - Deng et al. (2017) sought to identify long noncoding RNAs (lncRNAs) participating in hippocampal neurogenesis, and investigated their roles in this process. RNA obtained from whole hippocampi of adult Sprague-Dawley adult rats, both control and after fimbria-fornix (FF) transection, was hybridized with the RiboArray lncDETECT RAT Array and 74 upregulated and 29 downregulated lncRNAs were identified. Pathway analysis showed that the lncRNAs were involved in cell cycle and neurogenesis. In particular, among the upregulated lncRNAs, lncRNA2393 was shown to be expressed in the SGZ of the dentate gyrus and in the cytoplasm of NSCs (NSCs). Furthermore, the knockdown of lncRNA2393 depleted the EdU-positive stem/progenitor cells, suggesting that lncRNA2393 is part of the neurogenesis activation process.

*β-arr1* - Tao et al. (2015) explored the role of β-arrestin 1 (*β-arr1*) in adult mouse neurogenesis in the dentate gyrus. They showed that *β-arr1* is expressed both in dentate gyrus NSCs and neurons; furthermore, *β-arr1* knockout caused a decrease of proliferating progenitor cells, as well as NSCs (Nestin^+^GFAP^+^ cells), a defect that was not rescued by a neurogenic stimulus such as running. RNA-seq of primary cultures of *β-arr1* knockout dentate gyrus astrocytes revealed an upregulation of *Bmp2* and downregulation of *Shh*, *Il15*, and *Il17*. Since Bmp2 is a known antimitotic factor, its upregulation in *β-arr1* knockout could explain the observed decline in neurogenesis. Thus, β-arr1 regulates the production of excretive factors derived from niche astrocytes and expansion of neural precursors in DG.

*Yap1* - Fan et al. (2023) studied the effect of the transcriptional co-activator *Yap1* (Yes1 associated transcriptional regulator) on adult mouse NSC activity, starting from the observation of Hochgerner et al. (2018) that Yap1 is highly expressed in activated NSCs of adult dentate gyrus. Conditional knockout of *Yap1* with a *Glast-Cre* mouse caused a decrease of dentate gyrus proliferative NSCs, suggesting a role of Yap1 in NSC activation. This was consistent with single-cell RNA-seq data indicating that overexpression of a gain-of-function mutant of *Yap1* – which induces adult NSC proliferation *in vivo* - leads to a decrease in the expression of genes that are typically expressed in quiescent NSCs and an increase in genes linked to the cell cycle and NSC activation in tandem.

*α2-chimaerin* - Su et al. (2019) studied the role played by α2-chimaerin (*α2-C*), a Rho GTPase-activating protein, in the adult mouse hippocampal NSC homeostasis. When *α2-chimaerin* was conditionally deleted in adult NSCs, the cells proliferated less and prematurely differentiated into intermediate progenitor cells (IPCs), leading to eventual reduction of the NSC pool and compromised neuronal production. Then the authors isolated single hippocampal stem and progenitor cells by FACS followed by single-cell RNA-seq. They separated the data into different cell clusters based on the expression of standard marker genes. A distinct subpopulation of NSCs expressing the antiaging protein Klotho was found, but this subpopulation lacked in α2-chimaerin conditional knockout mice. This loss of Klotho expression occurred during the transition of NSCs to IPCs, according to single-cell RNA sequencing and pseudotime studies. The authors suggest that the depletion of Klotho-positive NSCs in *α2-C* knockout mice is the cause of early differentiation of NSCs and depletion of the pool. Authors conclude that α2-chimaerin plays a critical role in homeostasis of adult hippocampal NSCs and in maintaining neurogenesis.

*KDM4C*–Zhu et al. (2024) investigated the function of histone lysine demethylase 4C (*KDM4C*) in hippocampal NSCs. They showed that KDM4C, transduced by lentiviral vector either in primary hippocampal NSCs isolated from neonatal mice or *in vivo* in the dentate gyrus, increased the number of proliferating Ki67^+^/Sox2^+^ cells. By RNA-seq of isolated NSCs they identified genes regulated by KDM4C involved in development, cell cycle, and neurogenesis. Protein-protein interaction analysis (using STRING) of the top 10 up- and downregulated genes, revealed that KDM4C interacts with the ApoE protein and that the proliferative effect of KDM4C was inhibited following siRNA-mediated knock-down of ApoE. Thus, KDM4C acts through the ApoE protein.

*VEGF* - Microglia support adult hippocampal neurogenesis, as Kreisel et al. (2019) show. In fact, diphtheria toxin-induced microglia ablation in the mouse dentate gyrus reduced the survival of newly formed neuroblasts, resulting in impaired hippocampal neurogenesis. The authors also found that microglia residing in the dentate gyrus are specifically responsive to the neurogenic factor VEGF as microglia ablation impaired VEGF-induced neurogenesis. Furthermore, transcriptomic analysis uncovered a set of genes expressed in mouse dentate gyrus microglia responsible for the neurogenic response to VEGF. In particular the tyrosine kinase *Axl* was shown to be required for VEGF-induced neurogenesis increase. Thus, microglia residing in the dentate gyrus possess unique properties that support adult hippocampal neurogenesis.

- Neural differentiation

*IMPA1* - Figueiredo et al. (2021) sought to understand the role played by inositol monophosphatase 1 (*IMPA1*), a gene whose homozygous mutation in human causes intellectual disability. To this aim, they generated pluripotent NSCs (iPSCs) from patients and neurotypical controls and differentiated them into hippocampal dentate gyrus-like neurons and astrocytes. They showed that low-passage patient-derived NPCs presented cell cycle arrest, apoptosis and reduced neuronal differentiation. Interestingly, transcriptome analysis showed that in NPCs derived from *IMPA1*-mutated patients the most upregulated genes involved in cell cycle arrest were *p15INK4B* and *p16INK4A* while those linked to apoptosis were *CASP4* and *p16INK4A*. Additionally, there was prevalence of GO processes with upregulation of the gliogenic pathway and downregulation of neuronal differentiation, indicating the requirement of IMPA1 for these processes.

*Nfix* - Heng et al. (2014) showed that NPCs in the embryonic hippocampus express nuclear factor one X (*Nfix*), and that the differentiation of these progenitor cells is delayed in *Nfix*-null mice. Furthermore, postnatal *Nfix*-null animals displayed reduced length of the dentate gyrus, with aberrant migration and fewer NPCs in the developing SGZ (Prox1-positive cells). Microarray analysis of the whole embryonic (E16) hippocampus indicated upregulation of transcription factors (e.g., *Sox9*, *Sox5*, *Ngn2*) and genes involved in cell division and mitosis (Polo-like kinase 1 [*Plk1*], Protein regulator of cytokinesis 1 [*Prc1*], *cyclinD2*) and metabolism (Uracil DNA glycosylase [*Ung*]). Thus, Nfix is important for hippocampal morphogenesis.

*O-GlcNAc* - White et al. (2020) showed that the age-associated loss of the posttranslational modification O-linked β-*N*-acetylglucosamine (O-GlcNAc) in mouse hippocampal NSCs promotes a glial fate switch. Indeed, a correlation between age, loss of *O-GlcNAc*, and increased gliogenesis was detected in the adult hippocampus. Consistently, a decrease of O-GlcNAc induced by injecting a lentivirus expressing shRNA against *O-GlcNAc* transferase in dentate gyrus, impaired the production of DCX^+^ neuroblasts and NeuN^+^ neurons, while *in vitro* it promoted astrocyte differentiation. Through RNAseq of primary NSCs treated with O-GlcNAc transferase inhibitor they identified upregulated transcription factors associated with NSC function, including *STAT3*, a key inducer of astrocyte differentiation (He et al., 2005), and they confirmed the critical role of STAT3, since a gliogenic fate shift in NSCs was triggered by loss of O-GlcNAcylation of STAT3 at Threonine 717 (T717) in the hippocampus. Thus, O-GlcNAc prevents a gliogenic shift in NSCs.

### Dentate gyrus gene profiles in neurodegenerative diseases, neuroinflammation and other neural pathologies

There is wide evidence that altered neurogenesis is involved in the pathology of many neurodegenerative, neurodevelopmental, psychiatric and neuroinflammatory disorders.

- Neurodegenerative diseases are associated to neuroinflammation that affects adult hippocampal neurogenesis in various ways, with consequent impairment of cognitive functions. In fact, it is known that in Parkinson’s disease frequently occurs a decrease of neurogenesis and neuronal differentiation as a consequence of reduced dopaminergic activity, while in Alzheimer’s disease the neuroinflammation state causes either an increase of neurogenesis, e.g., through stimulation of microglia by Il-4 and Il-10, or a decrease of neurogenesis, e.g., in streptozotocin-induced neuroinflammation, as seen in mouse models (see for review Amanollahi et al., 2023). On the other hand, epilepsy is usually associated to increased neurogenesis accompanied by aberrant migration of progenitor cells in DG (Amanollahi et al., 2023).
- Schizophrenia is a mental illness with a major genetic component and interacting non-genetic factors, whereas autism spectrum disorder is a group of severe neurodevelopmental disorders affecting social behavior. Dysfunctions of neurogenesis have frequently been associated with schizophrenia and autism spectrum disorder, and it has been advanced the hypothesis that these dysfunctions may play a role in their etiology and development (Iannitelli et al., 2017; Chen et al., 2024). Likewise, social isolation causes mental illness, namely, depression and impairment of social memory, which appear to depend on reduced adult neurogenesis; in fact, neurogenic stimuli can rescue social memory deficits (Monteiro et al., 2014).
- Neuroinflammation can be caused by different immune components such as activated glia, chemokines, infectious diseases or immune/autoimmune disorders, and each of them has a different impact on each step of adult neurogenesis, either positive or negative depending on various factors, such as chronicity or severity of the inflammation (Amanollahi et al., 2023).

Here we review studies that have revealed transcriptomic changes occurring in the hippocampus or NSCs of models of neurodegenerative, mental health and neurodevelopmental disorders as well as neuroinflammation models associated with altered neurogenesis, such as spontaneously hypertensive rats, *TLX* knockout or HIV infected mice, and a Systemic lupus erythematosus mouse model.

See Supplementary Table S3 for summary.

- Parkinson’s disease

MPTP-induced Parkinsonian Syndrome - Bao et al. (2017) analyzed the transcriptome of the dentate gyrus and the SVZ of mice treated with MPTP (1-methyl-4-phenyl-1,2,3,6-tetrahydropyridine), a substance that destroys the dopaminergic nuclei of substantia nigra, thus mimicking Parkinson’s disease. Since dopaminergic denervation of dentate gyrus and SVZ impairs neurogenesis in the SGZ and in SVZ (Höglinger et al., 2004), it was relevant to assess the transcriptomic changes induced by MPTP treatment. It turned out that in the dentate gyrus occurred an upregulation of the transcriptional repressor *Hdac4* and a downregulation of *Fos* and *Nr4a1* as a consequence of inactivation of ERK signaling by MPTP. Indeed, it is known that inhibition of MAPK/ERK signaling worsens hippocampal neuronal apoptosis, inhibits neurogenesis and impairs cognitive performances (Jiang et al., 2015). Moreover, in mouse dentate gyrus 3 weeks after MPTP exposure, genes involved in neural plasticity were differentially expressed, with a significant reduction in the expression of insulin-like growth factor 2 (*Igf2*) which acts as positive regulator of glucose-mediated insulin secretion; its decrease is associated to a decrease of insulin secretion and an increase of fatty acid metabolism, a signal associated with inactivity of stem cells in the dentate gyrus (Shin et al., 2015). Different, sometimes opposite, transcriptional effects were observed in SVZ, indicating that MPTP-induced Parkinson’s disease induces niche-specific transcriptional profiles.

*LRRK2* - Schulz et al. (2011) sought to define the role of the leucine-rich repeat kinase 2 (*LRRK2*) gene, whose dominant mutations are a frequent cause of Parkinson’ disease. They show that *LRRK2* deficiency in mouse embryonic stem cells led to faster neuronal differentiation induced by retinoic acid. This was accompanied, as detected by microarray analysis of embryonic stem cells-derived neurons, by a decrease of pluripotency-associated genes, like *Nanog*, *Oct4*, and *Lin28*. Correspondingly an increase in the number of NPCs was observed in the hippocampal dentate gyrus of *LRRK2*-deficient mice (total DCX-positive cells). The authors conclude that at the origin of the faster differentiation is a modulation of the retinoic acid receptor signaling system by Parkinson’s disease-linked *LRRK2* mutations.

- Alzheimer’s disease (AD)

LY01 - Li et al. (2022) analyzed the effect on adult neurogenesis and NSC transcriptome of the treatment with Cytisine N-methylene-(5,7,4′-trihydroxy)-isoflavone (LY01), a compound isolated from the Chinese herbal medicine *Sophora alopecuroides,* in the 5 × Familial Alzheimer’s Disease (5 × FAD) mouse model of early AD. They found that LY01 treatment for 2–5 weeks increased the production of neuroblasts and immature neurons (DCX^+^ cells) in the dentate gyrus and, in parallel, promoted the proliferation and migration of primary NSCs from dentate gyrus. LY01 treatment also promoted associative memory performance in a fear conditioning task. Transcriptomic profiling by RNA sequencing of primary NSCs showed that 237 genes were differentially regulated by LY01. Gene Ontology analysis suggested that extracellular matrix (ECM) and associated receptors may play a role in the action of LY01. The most upregulated gene was *Lamc2* (Laminin subunit gamma-2), which encodes a component of the ECM. Consistently, it is known that laminin is the main glycoprotein of the ECM and promotes NSC regeneration (Ma et al., 2020).

ENT-A011 - Charou et al. (2024) demonstrated that ENT-A011, a novel agonist of the TrkB receptor, exerts a neurogenic and neuroprotective action in stem cell models of AD in a manner comparable to brain-derived neurotrophic factor (BDNF). Specifically, they showed that ENT-A011 was able to stimulate proliferation and prevent oligomeric amyloid-β-induced cell death in adult primary mouse hippocampal NSCs and NPCs derived from induced pluripotent stem cells (iPSC) from healthy and AD donors. Through RNA-seq of human AD iPSC-derived NPCs, they showed that ENT-A011 acts through the BDNF downstream gene network.

- Epilepsy

Valproic acid - Sakai et al. (2018), examined the effect of prenatal exposure to the anti-epileptic drug valproic acid (VPA) on adult mouse hippocampal neurogenesis and gene expression profile. They found that prenatal exposure to VPA causes altered migration of newborn neurons in the dentate gyrus, which are integrated into the granular layer of the dentate gyrus, but most of them are ectopically delocalized into the hilus, with consequent increase of seizure susceptibility. RNA-seq analyses showed that prenatal VPA treatment altered the expression in neural stem/progenitor cells of genes associated with cell migration. Authors identified deregulated genes in the dentate gyrus of mice at 12 weeks of age, enriched in migration-related GO terms, and in particular two downregulated genes, contactin 2 (*Cntn 2*) and CXC motif chemokine receptor 4 (*Cxcr4*). Cntn 2 modulates migration in embryonic brain (Denaxa et al., 2001), while Cxcr4 is necessary for correct positioning of newborn neurons in the adult hippocampus (Schultheiß et al., 2013). Authors showed that Cxcr4 downregulation was responsible for the ectopic migration, as restoration of Cxcr4 by a GFP-retrovirus in the dentate gyrus of 4-week-old mice, led to correct localization of new neurons (GFP/NeuN^+^) and decreased the seizures induced by Kainic acid. This shows that the role of Cxcr4 in the migration of newborn neurons has a wide impact on the onset of epilepsy and points to potential therapies.

KCl depolarization - Walker et al. (2012) analyzed by RNA microarray the whole genome of KCl-depolarized mouse primary adult hippocampal cells, a model of latent stem and progenitor cell activation mimicking the epileptic status *in vivo*. Authors found that *Wnt3* and Prolactin (*PRL*) were among the most upregulated genes in depolarized hippocampal cells and focused the study on the role of PRL in the hippocampus. Authors showed that exogenous PRL can increase the number of hippocampal precursors both *in vitro* and *in vivo*. Conversely, *PRL*-null mice showed 80 percent decrease in the number of hippocampal-derived neurospheres, without change in precursor cell proliferation *in vivo*, suggesting that other factors may compensate for the *PRL* loss. Interestingly, *PRL*-null mice displayed learning and memory deficits that were rescued by infusion of PRL into the hippocampus, thus indicating a role of this gene in hippocampal neurogenesis-associated cognitive functions.

*FosB* - Yutsudo et al. (2013) analyzed the role of FosB in epilepsy and depression, given that these two pathologies are correlated and that: i) adenovirus-mediated expression of *FosB* gene induces NSC proliferation in rat embryonic cortical cell cultures; ii) forebrain ischemia in the rat brain induces FosB expression in the dentate gyrus (Kurushima et al., 2005). Authors showed that *FosB* knockout mice exhibit depression and spontaneous epilepsy symptoms with ectopic migration of NPCs and reduced neurogenesis in the hippocampus. Hippocampal microarray analysis revealed that genes involved in neurogenesis, depression, and epilepsy were downregulated in the hippocampus of *FosB*-null mice (*VGF*, *Gal*, *Dlk1*, *Smad3*, *Trh*, *Penk*), indicating a specific role of FosB in depression and epilepsy, based on the defective adult hippocampal neurogenesis in *FosB*-null mice.

- Schizophrenia

iPS cells - Hathy et al. (2020) analyzed the gene expression profile of dentate gyrus NPCs generated from induced pluripotent stem cell (iPSC) lines obtained from a schizophrenia patient carrying *de novo* genetic mutations and his unaffected parents. iPSC lines were produced from peripheral blood mononuclear cells of a schizophrenia patient and his parents using Sendai virus-based reprogramming. These iPSCs were then differentiated into neural progenitor cells (NPCs) and hippocampal dentate gyrus granule cells. Then authors analyzed by RNA-seq the gene expression profile of the patient-derived NPC lines, compared to that of cell lines obtained from the patient’s father and mother. Authors found 273 genes upregulated in the patient’s NPCs vs the parental NPCs, including *AUTS2*, *ERBB4*, *GRIN2A*, and *KHDRBS2* which have been implicated in the etiology of schizophrenia. There was also an enrichment of GO terms of genes involved in neurogenesis, neuronal differentiation, as well as Hippo and Wnt signaling. This gene profile was accompanied by increased proliferation of the patient’s NPCs.

- Autism Spectrum Disorder

Fullerenols – Luo et al. (2024) studied the effect on hippocampal adult neurogenesis of the treatment for 1 week with the neuroprotective carbon nanomaterial fullerenols in BTBR mice, a model of Autism Spectrum Disorder (ASD). They observed an increase of neuroblasts (DCX^+^) and NSCs (Sox2^+^/GFAP^+^) in the dentate gyrus of BTBR mice after fullerenols supplementation. This corresponded to an increased performance in short-term memory tasks (novel object recognition) and spatial memory (Y maze). RNA-seq analysis of the isolated mouse hippocampus revealed that several genes related to neurogenesis were downregulated in BTBR mice but restored after fullerenols treatment and highlighted a critical role of VEGFA in mediating the therapeutic effect of fullerenols.

- Social isolation

Ibi et al. (2008) sought to study the effect of social isolation (SI) rearing after weaning on memory and dentate gyrus neurogenesis and to identify regulated genes. SI reduces in young mice the number of new neurons generated in the dentate gyrus (BrdU-positive cells), an effect due to an impaired survival of new neurons rather than to reduced proliferation of progenitor cells. SI also reduced spatial memory, an effect rescued by the antidepressant fluoxetine. By RNA microarray of isolated dentate gyrus, the authors identified four genes downregulated by SI, *Nurr1* and *Npas4*, *Arc* and *Fos*. *Npas4*, *Fos* and *Arc* are involved in GABAergic synaptic transmission (*Npas4*), dendritic spine morphogenesis (*Arc*), memory (*Npas4*, *Arc*), and learning (*Arc*, *Npas4*). The authors concluded that lack of social interaction at a young age impairs the process of hippocampal neurogenesis, which may play a role in the onset of mental illnesses.

- Hypertension

Spontaneously hypertensive rats (SHR) - Nyberg et al. (2005) studied hippocampal neurogenesis in spontaneously hypertensive rats (SHR), which show higher proliferation rates of cells in the dentate gyrus. By whole-hippocampus RNA array they identified the gene encoding the glucose-dependent insulinotropic polypeptide (*GIP*), whose expression correlates with cell proliferation rates in the adult rat dentate gyrus. Moreover, GIP infused intracerebroventricularly increased the proliferation of cells (BrdU^+^) in the granule cell layer of the dentate gyrus. This is evidence for a regulatory function of GIP in dentate gyrus neurogenesis.

- Hippocampal inflammation

*TLX* receptor - Ó'Léime et al. (2018) sought to identify the role in hippocampal inflammation of the orphan receptor TLX, which is highly expressed in hippocampal NPCs, where it stimulates proliferation. The authors compared by RNA-seq the transcriptional profile in the hippocampus of *TLX* knockout mice with the effects on gene expression induced by the pro-inflammatory cytokine IL-1β injected *in vivo* into the wild-type mouse hippocampus. IL-1β suppresses neural progenitor cell proliferation as well as TLX expression in the hippocampus. By whole-hippocampus RNA-seq they observed that the knockout of *TLX* upregulates 1272 genes, compared to wild-type, of which 1096 are implicated in cell chemotaxis (*Cxcr2*, *IL17RA*, *Cxcl10*, *Fgf2*, *Cxcl5*, *Ccl9*, *Ccr1*, *Cxcl13*) and 176 are in common with genes increased in wild-type mice injected with IL-1β into the hippocampus and are implicated in cell response to cytokine stimulus (*Nfkbia*, *IL6*, *Cxcl1*, *Ccl2*). In summary, genes implicated in the inflammatory process signaling, including TNF signaling (*Tnf*, *Fos*, *Jun*, *Il1b*), cytokine-receptor interaction (*Csf3*, *Il6*, *Ccl2*, *Il1b*, *Il1a*), and NF-κB signaling (*Nfkbia*, *Lbp*, *Ptgs2*), were significantly enriched upon *TLX* deletion. This indicates that *TLX* knockout mice exhibit a dysregulation of inflammatory genes similar to that elicited by IL-1β in the hippocampus of wild-type mice.

HIV-1 - HIV-1 infection causes neuroinflammation that reduces the survival of stem and progenitor cells due to toxic viral proteins (gp120 and tat). Therefore, Hill et al. (2019) analyzed the hippocampal inflammatory transcriptome of mice chronically infused into the hippocampus for 14 days with the HIV-1 viral proteins gp120 or tat. This treatment reduced the proliferation of stem and progenitor cell within the hippocampal SGZ as detected by DCX/BrdU or Ki67-positive cells. Analysis of inflammatory responses within the hippocampus by RNA-seq and Ingenuity Pathway Analysis indicated a significant upregulation of mRNA of several inflammatory genes such as *Il12a*, *Il6*, *Il1b*, *Tnf*, *Ccl2*, *Ccl4*, *Cxcl10* and the adhesion molecule *Icam1*. These data suggest that chronic administration of gp120 or tat induces an inflammatory condition that results in reduced hippocampal neurogenesis and cognition.

- Systemic lupus erythematosus (SLE)

NZB/W-F1 mice - Nikolopoulos et al. (2023) analyzed adult neurogenesis by immunohistochemistry, cognition tests and hippocampal RNA-seq in lupus-prone NZB/W-F1 mice. The autoimmune disorder known as systemic lupus erythematosus (SLE) is characterized by an excess of several pathogenic autoantibodies. B-cell hyperactivity, polyclonal hypergammaglobulinemia, and glomerulonephritis (immune complex deposition) are its hallmarks. The authors found behavioral abnormalities related to hippocampal function in lupus-prone mice at prenephritic stage. Disruption of hippocampal neurogenesis was the cause of this phenotype, as evidenced by increased proliferation, reduced differentiation, and increased apoptosis in hippocampal stem cells *ex vivo* and *in vivo*, in addition to microglia activation. Comparing nephritic to prenephritic lupus hippocampal tissue by RNA-seq, gene set enrichment analysis showed a significant inflammatory response, and the authors identify *IL-6* and *IL-18* as cytokines that directly induce apoptosis of adult hippocampal stem cells, thus being at the origin of the defective neurogenesis, cognition and inflammation.

## Discussion and conclusion

We have summarized transcriptomic studies focusing on hippocampal neurogenesis in both primates and mouse*,* conducted *in vivo* or in cell models. These studies have highlighted interspecies differences, although the developmental mechanisms remain conserved.

Single-cell transcriptomic analyses of the adult dentate gyrus in primates (Hao et al., 2022; Zhang et al., 2021; Wang et al., 2022) and humans (Zhou et al., 2022; Simard et al., 2024) have confirmed the existence of imGCs and demonstrated that progenitor cells arise from adult NSCs. Notably, imGCs are more abundant in primates than in mice (Hao et al., 2022; Zhang et al., 2021), and in the human adult hippocampus they are generated *de novo* with low frequency and protracted maturation from stem/progenitor cells (Zhou et al., 2022). ImGCs are thought to play a significant role in the adult hippocampal plasticity, possibly by encoding new memories. Additionally, there are 29 primate-specific genes in NSCs (Wang et al., 2022) and 15% of human-specific genes in imGCs that are not detected in mouse (Zhou et al., 2022), thus highlighting interspecies differences, despite the preservation of developmental mechanisms (Zhong et al., 2020).

The transcriptomic landscape of neurogenesis in the mouse dentate gyrus has been extensively studied across different developmental stages and during aging, offering insights into the dynamic transitions between quiescent and activated neural stem cells (NSCs). Hochgerner et al. (2018) proposed a model of perinatal and adult neurogenesis with well-defined cell types and transitions, and with a long-lived population of NSCs in quiescent state but able to become activated and proliferating, and with quiescent-state genes activated. In contrast, Berg et al. (2019), by clonal lineage analysis based on the gene Hopx, proposed a continuous model of neurogenesis extending from embryonic stages to adulthood. Shin et al. (2015) focused on the transcriptional trajectories of quiescent and activated NSCs in adult mouse hippocampus, finding that the onset of NSC activation is coincident with the downregulation of Notch signaling, GABA and BMP pathways, fatty acid, sphingolipid and glutathione metabolism, and of some glycolysis genes. Borrett et al. (2022) identified 94 mRNAs upregulated in SVZ and SGZ NSCs during the postnatal transition to quiescence and subsequently downregulated when cells are reactivated to produce adult-born offspring, reacquiring a development-like state. Thus, NSC populations in the dentate gyrus and in the SVZ are transcriptionally similar throughout their lifespans despite the different types of neurons they generate.

**FIGURE 1:**
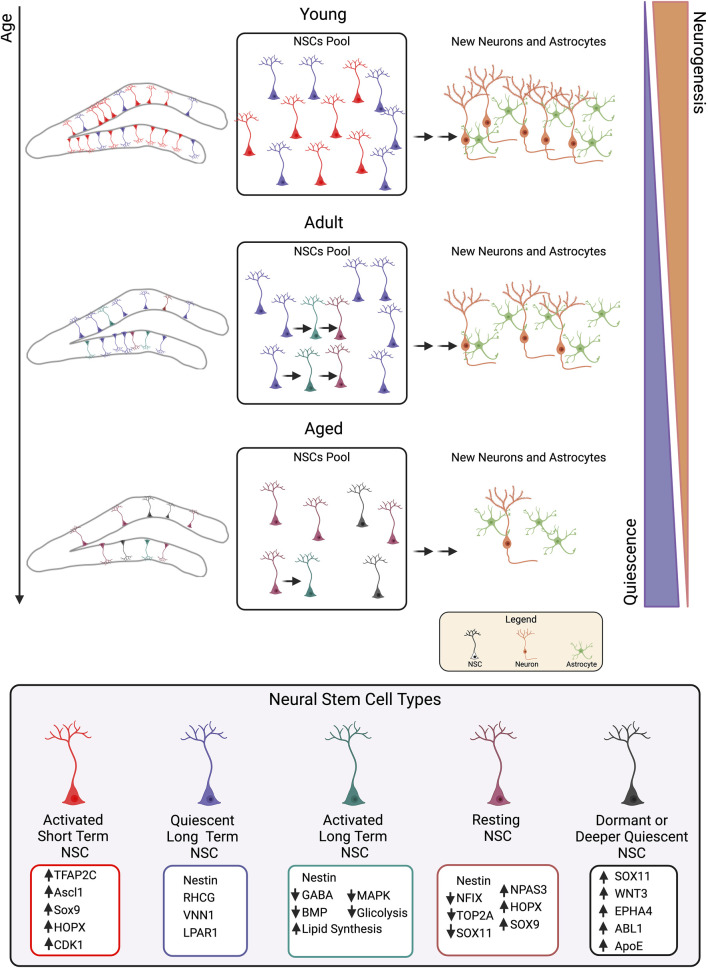
Representation of dentate gryus neural stem cell self-renewal dynamics. According to Harris et al. (2021) and Ibrayeva et al. (2021), single-cell RNA-seq and clonal analysis show that, in early postnatal stages, the “disposable” model of NSCs is prevalent, leading to rapid replication and severe reduction of the pool. However, during aging and later stages, NSCs gradually self-renew, preserving the pool through a gradual pattern. This age-related change coincides with a transition from frequent division to a more quiescent population. Ibrayeva et al.'s (2021) research demonstrates that there are heterogeneous populations of NSCs, with nestin-positive NSCs having a longer lifespan than those expressing the pro-activation gene Ascl1, due to reduced division and increased quiescent cells. The main genes involved in each NSC state are indicated, according to Hochgerner et al. (2018), Jimenez-Cyrus et al. (2024), Shin et al. (2015), Berg et al. (2019), Ibrayeva et al. (2021), and Borrett et al. (2022). Figure created with BioRender (http:biorender.com/).

Ibrayeva et al. (2021) demonstrated the presence of heterogeneous populations of NSCs in adult mouse hippocampus: a short-term Ascl1-labeled subpopulation, and a Nestin-labeled subpopulation exhibiting long-term viability. They observed a decrease in the number of dividing Nestin^+^ NSCs with aging and in parallel an increase in the percentage of NSCs in quiescence. This suggests that aging does not lead to clonal depletion but rather to a transition toward a population of quiescent NSCs. See Figure 1.

Of note, there are examples of the possibility to reactivate resting or dormant NSCs by a neurogenic stimulus (e.g., Micheli et al., 2021; Micheli et al., 2023), thus suggesting that NSC self-renewal is a highly plastic process.

Moreover, several studies have assessed by RNA-seq the effects of the deregulation of individual genes implicated in hippocampal neurogenesis, revealing their impact on NSC function, e.g., *Arid1a* (Liu et al., 2023), *Sox9* (Caramello et al., 2022), *Fasn* (Knobloch et al., 2013) or *KDM4C* (Zhu et al., 2024). Similarly, several transcriptomic investigations on specific genes or substances involved in neurodegenerative diseases and affecting neurogenesis, e.g., the leucine-rich repeat kinase 2 (*LRRK2*) gene favoring Parkinson’s disease (Schulz et al., 2011), or the neuroprotective TrkB agonist ENT-A011 (Charou et al., 2024) have highlighted their impact on NSC function, offering insights into potential therapeutic approaches.

## Glossary

AD: Alzheimer’s disease
AI: artificial intelligence
ApoE: apolipoprotein E
Ascl1: Achaete-Scute bHLH factor 1
ASD: Autism Spectrum Disorder
BBB: blood brain barrier
BDNF: brain-derived neurotrophic factor
BrdU: 5-bromo-2′-deoxyuridine
CAS: core aging signature
Cxcr4: CXC motif chemokine receptor 4
DCX: Doublecortin
DG: dentate gyrus
E: embryonic day
EED: embryonic ectoderm development
ETNPPL: ethanol phosphate phospholipase
FACS: fluorescence-activated cell sorting
GC: granule cells
GFAP: glial fibrillary acidic protein
GFP: green fluorescent protein
GO terms: Gene Ontology terms
Hopx: Homeodomain-only protein
IFN-gamma: Interferon-gamma
imGC: immature GC
IMPA1: inositol monophosphatase 1
IP: intermediate progenitor
IPs: intermediate progenitors
iPSC: induced pluripotent stem cell
KA: kainic acid
KDM4C: histone lysine demethylase 4C
L-RUN, H-RUN: low or high runners
lncRNAs: long noncoding RNAs
LPA1: lysophosphatidic acid receptor 1
LRRK2: leucine-rich repeat kinase 2
Mbd1: methyl-CpG-binding domain 1
MPTP: 1-methyl-4-phenyl-1,2,3,6-tetrahydropyridine
MTLE: mesial temporal lobe epilepsy
Nfix: nuclear factor one X
NPCs: neural progenitor cells
NSC: neural stem cell
O-GlcNAc: O-linked β-N-acetylglucosamine
P: postnatal day
PRL: Prolactin
Prox1: Prospero homeobox 1
REST: Repressor element 1-silencing transcription factor
RGL: radial glial like
sc/snRNA-seq: single-cell/single-nucleus RNA Sequencing
SGZ: subgranular zone
SHR: spontaneously hypertensive rats
SI: social isolation
Sirt1: Sirtuin 1
Snca: alpha-synuclein
Sox2: Sex determining region Y-Box 2
SVZ: subventricular zone
TAPC: transiently amplifying progenitor cell
TBI: traumatic brain injury
VPA: valproic acid.
