# Supplementary material for: Survey of transcriptome analyses of hippocampal neurogenesis with focus on adult dentate gyrus stem cells
Source: Front Cell Dev Biol. 2025 May 30;13:1605116. doi: 10.3389/fcell.2025.1605116 (PMC12162651; doi:10.3389/fcell.2025.1605116)
Supplement: Supplementary file 1 [file Table2.docx]

**Table S2. Effect of the deregulation of specific genes on dentate gyrus NSC neurogenesis and transcription**

| **Gene and aim** | **Model/methods** | **Key findings and DE genes** | **Reference** |
| --- | --- | --- | --- |
| **NSC stemness/self-renewal** | | | |
| ***EED*** (embryonic ectoderm development), core component of the Polycomb complex. Ascertaining its role in neurogenesis | RNA-seq of DG of P14 *EED*-KO or WT. By crossing *EED^f/f^* mice with Tg hGFAP-*Cre*, *EED* was deleted in NSPCs from E13.5 | *EED* conditional KO has impaired neuronal differentiation and malformation of DG.  561 up-regulated and 486 down-regulated genes, involved in neuronal development and differentiation. Best candidate: *Prox1* and *Sox11* (down-regulated) and *p16Ink4a* (up-regulated) | *Liu et al., 2019* |
| ***LPA1*** (Lysophosphatidic acid receptor 1). Study of activator role of lipidogenesis on neurogenesis | RNA seq of LPA1-GFP^+^/EGFR^+^/prominin^+^ cells sorted by FACS (proliferating NSCs/NPCs) | The transcriptomic profile of LPA1^+^ proliferating NSCs of DG includes genes involved in cytokine signaling, suggesting a cross talk between NSCs and immune system | *Walker et al., 2016* |
| ***FoxO3*** (Forkhead boxO3)  Exploration of pathways activated in DG and forebrain | Microarray of RNA from forebrains of Tg *FoxO3* mice.  (tet-off Tg mouse expressing *FOXO3* by *CamKII* promoter in forebrain, DG neuroblasts and neurons) | Overexpression of *FoxO3* causes decrease of brain size, included DG, and enhanced apoptosis.  *Pik3ip1* is a FoxO3 target gene that may enhance preexisting proapoptotic stimuli. | *Schmidt-Strassburger et al., 2012* |
| ***Mbd1*** (methyl-CpG-binding domain 1).  Ascertaining its role in neurogenesis | RNA seq of Nestin^+^GFP cells FACS isolated from adult DG (8-week-old) | *Mbd1* is important for the neurogenic potency and the integrity of DG NSCs.  Enrichment of upregulated astrocytic genes and of down-regulated neuronal genes | *Jobe et al., 2017* |
| ***Sirt1*** (Sirtuin 1) in the control of hippocampal adult neurogenesis | Microarray analysis of Sirt1 KO or wild-type neurospheres resveratrol-treated | Sirt1 appears to operate as an endogenous negative control on "stemness" genes activator of NSC self-renewal.  Identified genes regulated in *Sirt1* KO and counter-regulated by pro-neurogenic resveratrol: metabolic pathways and neurogenesis genes (*EphA4*, *Sox7*, *Sox10*, *Fgfr3* and *Erbb4*) | *Ma et al., 2014* |
| ***Sox2*** and ***DCX*** (Doublecortin). Comparison of expression signatures of NSCs and of NPC/imGC of DG | Microarray of NSCs (Sox2^+^) and progenitor cells/immature neurons (DCX^+^) isolated by cell sorting of DG, from mice expressing GFP via *Sox2* promoter or DsRed via *Dcx* promoter, respectively | *Igf2* is highly expressed in NSCs (Sox2^+^) and stimulates the proliferation of cultured DG NSCs. Sox2 cells: enriched in GO terms associated with cell differentiation and generation of energy, and lipid metabolic process. DCX cells: enriched in neurogenesis and generation of neurons | *Bracko et al., 2012* |
| **NSC quiescence** |  |  |  |
| ***Sox9*** (SRY-Box Transcription Factor 9).  Identification of its role in development of NSCs of DG | RNA-seq of archicortex (primitive DG) of mice with conditional deletion of Sox9 in archicortex | *Sox9* and *Hopx* are highly expressed in the primitive DG (cortical hem) but do not confer NSC potential, suggesting that these genes contribute to astrocytic differentiation | *Caramello et al., 2022* |
| ***REST*** (RE1 Silencing Transcription Factor). Investigating its role in the mechanisms controlling adult NSCs self-renewal | RNA seq of cultured hippocampal NSCs electroporated with a *REST* shRNA to knockdown *REST* | REST is required to maintain NSCs/NPCs quiescence to prevent premature differentiation. *REST* KO progenitor cells show an increased differentiation gene profile. A superimposed Chip-seq identifies cell cycle genes unique to Ki67^+^ *REST* KO cells (*Cdc20*, *Cdk5r2*, *Tipin*), indicating a role in NSCs quiescence maintenance | *Mukherjee et al., 2016* |
| ***TGF-β1***. Study on effect of its overexpression on hippocampal neurogenesis | Microarray analysis of rat hippocampal neurospheres of a transgenic mouse conditionally expressing TGF-β1 in the hippocampal NSCs | TGF-β1 inhibits neural proliferation and induces NSC quiescence and neuronal survival. TGF-β1 is present in high levels in ageing and in neurodegenerative diseases.  Smad pathway is activated, and cell cycle genes (*cyclin G1*, *E*, *D2* and *B1*, *p57*) are regulated. Furthermore TGF-β1 induces neurogenesis-associated genes such as *Ascl1*, the Notch pathway gene *HES1*, the Notch ligands *Dll1* and *Jag1* | *Kandasamy et al., 2014* |
| **NSC and NPC proliferation/activation** | | | |
| ***Arid1a*** (AT-rich interactive domain-containing protein 1A), SWI/SNF chromatin-remodeling gene.  Ascertaining its role in DG adult neurogenesis | scRNA-seq of hippocampus from mouse with conditional deletion of *Arid1a* in cortex and hippocampus | *Arid1a* is a gene required for adult neurogenesis in the DG. In *Arid1a* KO hippocampus, *Prox1* is downregulated; stem/progenitor cells, neuroblasts and imGCs all decrease | *Liu et al., 2023* |
| ***Fasn*** (Fatty Acid Synthase) enzyme in *de novo* lipogenesis | Microarray of  Spot14-negative and -positive cells, isolated by cell sorting from a knockout Spot14-*Cre*ERT2 mouse | *De novo* Fasn-dependent lipogenesis is required for NSCs/NPCs neurogenesis activation.  NSCs Spot14-negative, which present higher proliferation and *de novo* lipidogenesis, have enrichment of upregulated genes in GO terms such as cell cycle and DNA replication | *Knobloch et al., 2013* |
| **LncRNAs** (Long noncoding RNAs). Participation to hippocampal neurogenesis | Microarray from whole denervated hippocampi of adult Sprague- Dawley rats, for lncRNA expression by RiboArray lncDETECT RAT | LncRNA2393 is expressed in NSC/NPCs of DG and is part of the process of neurogenesis activation. Authors found 74 up-regulated and 29 down-regulated lncRNAs in fimbria-fornix denervated hippocampi, involved in cell cycle and neurogenesis, with up-regulated lncRNA2393 in DG NSCs. | *Deng et al, 2017* |
| ***β -arr1*** (β-arrestin1). Investigation of participation to adult DG neurogenesis | RNA-seq of RNA extracted from primary niche astrocytes cultured from DG of WT and *β -arr1* KO mice | β -arr1 is expressed in DG in NSCs and neurons and is required for adult hippocampal neurogenesis. The study reveals that *Bmp2*, an antimitotic factor, is up-regulated in primary cultures of *β-arr1* KO DG astrocytes, while *Shh*, *Il15* and *Il17* are down-regulated, possibly explaining the observed decline of neurogenesis | *Tao et al., 2015* |
| ***Yap1*** (Yes1 associated transcriptional regulator)**.**  Studying its role in NSCs quiescence | scRNA-seq of cells expressing the GFAP-*Yap1* IRES EGFP lentivirus injected in DG, then isolated by FACS | The conditional deletion of *Yap1* in a Glast-*Cre* mouse indicates a decrease of DG proliferative NSCs, suggesting a role of Yap1 in NSCs activation.  Overexpression of Yap1 leads to a decrease in the expression of genes that are typically expressed in quiescent NSCs and a rise in genes linked to the cell cycle and NSC activation. | *Fan et al., 2023* |
| ***α2-C*** (α2-chimaerin), a Rho GTPase-activating protein. Studying its role in the homeostasis of adult hippocampal NSCs | scRNAseq of Nestin^+^YFP^+^ FACS isolated adult DG cells | α2-C plays a critical role in homeostasis of adult hippocampal NSCs.  Adult NSCs *α2-C* KO proliferate less and prematurely differentiate. scRNA-seq revealed a NSC subpopulation expressing the antiaging protein Klotho that lacked in *α2-C* KO mice, suggesting its role in precocious differentiation | *Su et al., 2019* |
| ***KDM4C*** (histone lysine demethylase 4C). Investigating its function on hippocampal NSCs | RNA-seq of isolated NSCs transduced by lentiviral overexpressing *KDM4C* | KDM4C promotes NSCs proliferation through modulation of ApoE. Top upregulated genes by KDM4C in NSCs: *ApoE* and *Slc1a3*; top downregulated genes: *Fos*, *Egr1*, *Cdkn1c* and *Sox10* | *Zhu et al., 2024* |
| ***Cx3cr1*** (markers of microglia) and ***VEGF*** (Vascular-Endothelial Growth Factor). Investigating their role on microglia as neurogenesis inducer | RNA-seq of microglia cells, from dissected DG, isolated by FACS of *Cx3cr1*-GFP Tg | Microglia residing in DG has unique properties in support of adult hippocampal neurogenesis. The tyrosine kinase Axl is required for the increase of dentate gyrus neurogenesis by VEGF | *Kreisel et al., 2019* |
| **Neural differentiation** | | | |
| ***IMPA1*** (Inositol monophosphatase 1), whose homozygous mutation in human causes intellectual disability | RNA-seq of iPSCs from patients differentiated into hippocampal DG-like neurons and astrocytes | *IMPA1* mutation upregulates gliogenic pathway and downregulates neuronal differentiation, indicating that these functions require IMPA1. In *IMPA1*-mutated patients' NPCs: up-regulation of cell cycle arrest (*p15Ink4b* and *p16Ink4a*) and apoptosis (*Casp4* and *p16Ink4a*) genes | *Figueiredo et al., 2021* |
| ***Nfix*** (nuclear factor one X). Studing its role in the embryonic and adult hippocampus | Microarray analysis of the whole *Nfix*-KO embryonic (E16) hippocampus | *Nfix* is important for hippocampal morphogenesis.  In *Nfix*-KO embryonic hippocampus are observed fewer SGZ NPCs and up-regulation of transcription factors (*Sox9*, *Sox5*, *Ngn2*) of genes involved in cell division and mitosis (*Plk1*, *Prc1*, *cyclinD2*) and metabolism (*Ung*) | *Heng et al., 2014* |
| Age-associated loss of ***O-GlcNAc*** (O-linked β-N acetylglucosamine). Effect on glial fate in NSCs | RNAseq of primary hippocampal NSCs treated with O-GlcNAc transferase inhibitor | The posttranslational modification O-GlcNAc prevents a gliogenic shift in NSCs. Identification of transcription factors associated with NSC function, including *STAT3*, a key inducer of astrocyte differentiation | *White et al., 2020* |

Abbreviations: DG: dentate gyrus; ImGC: immature granule cells; NSC: neural stem cell; scRNA-seq: single-cell RNA Sequencing.
