# Supplementary material for: Survey of transcriptome analyses of hippocampal neurogenesis with focus on adult dentate gyrus stem cells
Source: Front Cell Dev Biol. 2025 May 30;13:1605116. doi: 10.3389/fcell.2025.1605116 (PMC12162651; doi:10.3389/fcell.2025.1605116)
Supplement: Supplementary file 2 [file Table3.docx]

**Table S3. Dentate gyrus gene profiles in neurodegenerative diseases, neuroinflammation and other neural pathologies**

| **Neural pathology/Aim** | **Conclusion/key findings** | **Genes/cell types** | **Model/methods** | **Reference** |  |
| --- | --- | --- | --- | --- | --- |
| **Parkinson’s disease (PD)** | | | | |  |
| ***MPTP-induced Parkinsonian Syndrome.*** 1-methyl-4-phenyl-1,2,3,6-tetrahydropyridine, dopaminergic toxin inducing PD. Analysis of DG and V-SVZ of MPTP-treated mice | Down-regulation of *Igf2*, *Igfbp4* causes decrease of insulin secretion and increase of fatty acid metabolism, with possible inactivation of NSCs | Up-regulation of the transcriptional repressor *Hdac4* and down-regulation of *Fos* and *Nr4a1* as a consequence of the inactivation of ERK signaling by MPTP. Inhibition of insulin‑like growth factor 2 (Igf2), insulin-like growth factor binding protein 4 (*Igfbp4*) | RNA-seq of total RNA extracted from V-SVZ and DG of MPTP-treated mice | *Bao et al., 2017* |  |
| ***LRRK2*** (leucine-rich repeat kinase 2)-***KO mice***. Dominant mutations of *LRRK2* are frequent cause of PD; *LRRK2* role in NSCs function | Faster differentiation of DCX-positive cells in DG of *LRRK2*-KO mice because of modulation of the retinoic acid receptors signaling | Increase of the number of DCX^+^ cells in DG of *LRRK2*-KO mice with a decrease of pluripotency-associated genes (*Nanog*, *Oct4*, and *Lin28*) and increase of *Snca*, *Syn2*, *Slc6a11* | Microarray analysis of Embryonic stem cells differentiated into neurons with retinoic acid | *Schulz et al., 2011* |  |
| **Alzheimer’s Disease (AD)** | | | | |  |
| ***LY01-*** cytisine N-methylene-(5,7,4'-trihydroxy)-isoflavone (Chinese herbal *Sophora alopecuroides*). Studying the effect on ***5×Familial AD*** (5×FAD), models of early AD | LY01 promotes neural regeneration in the DG | 237 genes differentially regulated by LY01. Extracellular matrix (ECM) and associated receptors may play a role in the action of LY01. The most upregulated gene was *Lamc2*, ECM component | RNA seq of primary NSCs of DG of 5×FAD adult mice and treated with LY01 | *Li et al., 2022* |  |
| ***ENT-A011***, agonist of receptor TrkB. Investigating its role in neuroprotection and NSCs activity in iPSC of AD donors | ENT-A011 has neurogenic and neuroprotective activity as BDNF, being able to stimulate the proliferation of mouse primary adult DG NSCs and to reduce Aβ-induced cell death | ENT-A011 acts through the same gene network as BDNF in human NPCs | RNA-seq of mouse primary adult hippocampal NSCs and of human iPSC from AD donors | *Charou et al., 2024* |  |
| **Epilepsy** | | | | |  |
| ***Valproic*** and ***kainic acid*** (antiepileptic drugs). Investigating the effect on adult neurogenesis and on the gene profile | Prenatal antiepileptic drug exposure induces ectopic neurogenesis and augments seizure susceptibility in adult mice. Cxcr4, acting on newborn neuron migration, has a wide impact on epilepsy onset and points to potential therapies | Deregulated genes, in the NSCs/NPCs at 12 weeks, are enriched in migration-related GO terms, as contactin 2 (*Cntn 2*; modulates migration in embryonic brain, Denaxa et al., 2001) and *Cxcr4* (necessary for correct positioning of newborn neurons in adult hippocampus, Schultheiß et al., 2013) | RNA-seq of FACS-isolated NSCs/NPCs from DG of Nestin/EGFP mouse (E15, P5,12 weeks) prenatally exposed to VPA | *Sakai et al., 2018* |  |
| ***KCl depolarization*** of primary adult hippocampal cells: model of latent NSC/NPCs activation mimicking the epileptic status *in vivo* | Prolactin (PRL) loss results in learning and memory deficits in the *PRL*-KO mice, indicating a role of this gene in hippocampal neurogenesis also in functional terms | Genes most upregulated in depolarized hippocampi: *Wnt3* and *PRL*, which was chosen for analysis of its role in the hippocampus | Microarray analysis of RNA of KCl-depolarized whole mouse hippocampi | *Walker et al., 2012* |  |
| **FosB**.  Analysis of its the role in epilepsy and depression | Identification of a specific role of FosB in depression and epilepsy, based on defective adult hippocampal neurogenesis in *FosB*-KO mice | Genes involved in neurogenesis, depression, and epilepsy are down-regulated in the hippocampus of *FosB*-KO mice (*VGF*, *Gal*, *Dlk1*, *Smad3*, *Trh*, *Penk*) | Microarray analysis of whole hippocampus from adult FosB-KO mice | *Yutsudo et al., 2013* |  |
| **Schizophrenia (SZ)** | | | | |  |
| NPCs generated from ***iPSC*** lines obtained from a schizophrenia patient | Increased proliferation of iPSC-derived DG NPCs and upregulation of some gene implicated in schizophrenia etiology | 273 genes upregulated in NPCs from patient relative to parent NPCs, and in particular *AUTS2*, *ERBB4*, *GRIN2A*, and *KHDRBS2*, implicated in the etiology of SZ. There was also enrichment of GO involved in neurogenesis, neuronal differentiation, Hippo and Wnt signaling | RNA-seq of NPCs from iPSC lines from a schizophrenia patient | *Hathy et al., 2020* | |
| **Autism Spectrum Disorder (ASD)** | | | | | |
| **Fullerenols**, neuroprotective carbon nanomaterial in BTBR mice, an ASD model. Study of the effect on adult hippocampal neurogenesis | Rescue by fullerenols in the DG of BTBR mice of the decrease of neuroblasts (DCX^+^) and of NSCs (Sox2^+^/GFAP^+^) | VEGFA was involved in the rescue of hippocampal neurogenesis by fullerenols treatment | RNA-seq analysis of full isolated mouse hippocampus | *Luo et al., 2024* | |
| **Social isolation (SI)** | | | | | |
| ***SI*** rearing after weaning. Study of the effect on memory and DG neurogenesis | Lack of social interaction in young age damages the hippocampal neurogenesis process, which may play a role in the emergence of mental illnesses | Identification of genes downregulated by SI, which are involved in GABAergic synaptic transmission (*Npas4*), dendritic spine morphogenesis (*Arc*), memory (*Npas4*, *Arc*), learning (*Arc*, *Npas4*) | Microarray on RNA from isolated DG of SI and control mice | *Ibi et al., 2008* | |
| **Hypertension** | | | | |  |
| ***Spontaneously hypertensive rats (SHR***), showing increased proliferation of DG cells | Identification of a regulatory function for glucose-dependent insulinotropic polypeptide (*GIP*) in DG neurogenesis. GIP infused intracerebroventricularly increases cell proliferation in DG | Identification of *GIP*, whose expression correlated with cell-proliferation in adult rat DG. | RNA array of the whole mouse hippocampus | *Nyberg et al., 2005* |  |
| **Hippocampal inflammation** | | | | |  |
| Role of the ***Orphan receptor TLX***, expressed in hippocampal NPCs, where stimulates proliferation | In *TLX* -KO knockout mice there is a dysregulation of inflammatory genes similar to that elicited by IL-1 β in the hippocampus of WT mice | Genes implicated in the inflammatory process signaling, such as TNF signaling (*Tnf,* *Fos*, *Jun*, *Il1b*), cytokine-receptor interaction (*Csf3*, *Il6*, *Ccl2*, *Il1b*, *Il1a*), and NF-kB signaling (*Nfkbia*, *Lbp*, *Ptgs2*), were enriched upon *TLX* deletion | RNA- seq of the whole hippocampus of *TLX*-KO mice | *Ó'Léime et al., 2018* |  |
| ***HIV-1*** ***infection*** in mouse hippocampus. Study of the effect on inflammation and neurogenesis | Chronic administration of gp120 or tat induces an inflammatory condition that results in reduced hippocampal neurogenesis and cognition | Analysis of inflammatory responses within the hippocampus by RNA-seq and Ingenuity Pathway Analysis indicates a significant upregulation of several inflammatory genes such as *Il6*, *Il1b*, *Tnf*, *Ccl2*, and *Cxcl10* | RNA-seq and Ingenuity Pathway Analysis of genes expressed in hippocampus of mice chronically infused (14 days) with viral proteins HIV-1 gp120 or tat | *Hill et al., 2019* |  |
| **Systemic lupus erythematosus (SLE)** | | | | |  |
| ***NZB/W-F1*** lupus-prone mice. Investigating its role in neurogenesis | Identification of IL-6 and IL-18 as cytokines directly inducing apoptosis of adult DG NSCs, at the origin of the defective neurogenesis, cognition and inflammation | Comparing the lupus hippocampal tissue at the nephritic stage to the prenephritic stage by RNA seq, gene set enrichment analysis showed a significant inflammatory response | RNA seq of whole hippocampus | *Nikolopoulos et al., 2023* |  |

Abbreviations: DG: dentate gyrus; iPSC: induced pluripotent stem cell; NSC: neural stem cell; V-SVZ: ventricular-subventricular zone.
